# Specificity, frequency, and phenotype of citrullinated-specific T cells vary with disease activity in rheumatoid arthritis

**DOI:** 10.1172/jci.insight.202159

**Published:** 2026-09-08

**Authors:** Cliff Rims, Hannah A. DeBerg, Sylvia E. Posso, Virginia S. Muir, Hannes Uchtenhagen, Anne M. Hocking, Heather Bukiri, Jeffrey Carlin, Bernard Ng, Peter S. Linsley, Eddie A. James, Jane H. Buckner

**Affiliations:** 1 Center for Translational Immunology, and; 2 Center for Systems Immunology, Benaroya Research Institute at Virginia Mason, Seattle, Washington, USA.; 3 Department of Rheumatology, Virginia Mason Medical Center, Seattle Washington, USA.; 4 Rheumatology Section, VA Puget Sound Health Care System, Seattle, Washington, USA.; 5 Department of Medicine, Division of Rheumatology, University of Washington, Seattle, Washington, USA.

**Keywords:** Autoimmunity, Immunology, Autoimmune diseases, Rheumatology, T cells

## Abstract

In rheumatoid arthritis (RA), CD4^+^ T cells specific for citrullinated antigens (cit-antigens) are key drivers of disease, but knowledge about epitopes and phenotypes remains limited. We characterized the frequency and phenotype of cit-specific CD4^+^ T cells in peripheral blood using HLA class II tetramers combined with computational analysis of phenotypic clusters to simultaneously detect peptides derived from 5 cit-antigens (aggrecan, vimentin, fibrinogen, cartilage intermediate layer protein, and α-enolase) previously implicated in RA pathogenesis. In a cross-sectional cohort, cit-aggrecan–, cit-vimentin–, and cit-fibrinogen–specific T cells were more frequent in participants with RA than healthy volunteers, associated with active disease, and had Th1-like and stem-like lineages in RA. In a longitudinal cohort investigating response to therapy, the frequency of cit-aggrecan–, cit-vimentin–, and cit-fibrinogen–specific CD4^+^ T cells was significantly higher at baseline and further elevated in responders. Furthermore, the frequency of cit-specific Th1-like cells in responders decreased over time. In contrast, the frequency of Th1-like cells in non-responders increased over time. Collectively, these findings demonstrate that cit-specific CD4^+^ T cells are expanded in RA and target a broad number of antigens across a breadth of phenotypes. Furthermore, the predominant antigen specificities associate with disease activity and exhibit dynamic changes in phenotype that reflect response to therapy.

## Abstract 2

This study identified T cells that target several proteins in the joint, and linked them to disease activity in patients with rheumatoid arthritis. It also showed these cells change with treatment, suggesting potential markers for monitoring response to therapy.

## Graphical abstract

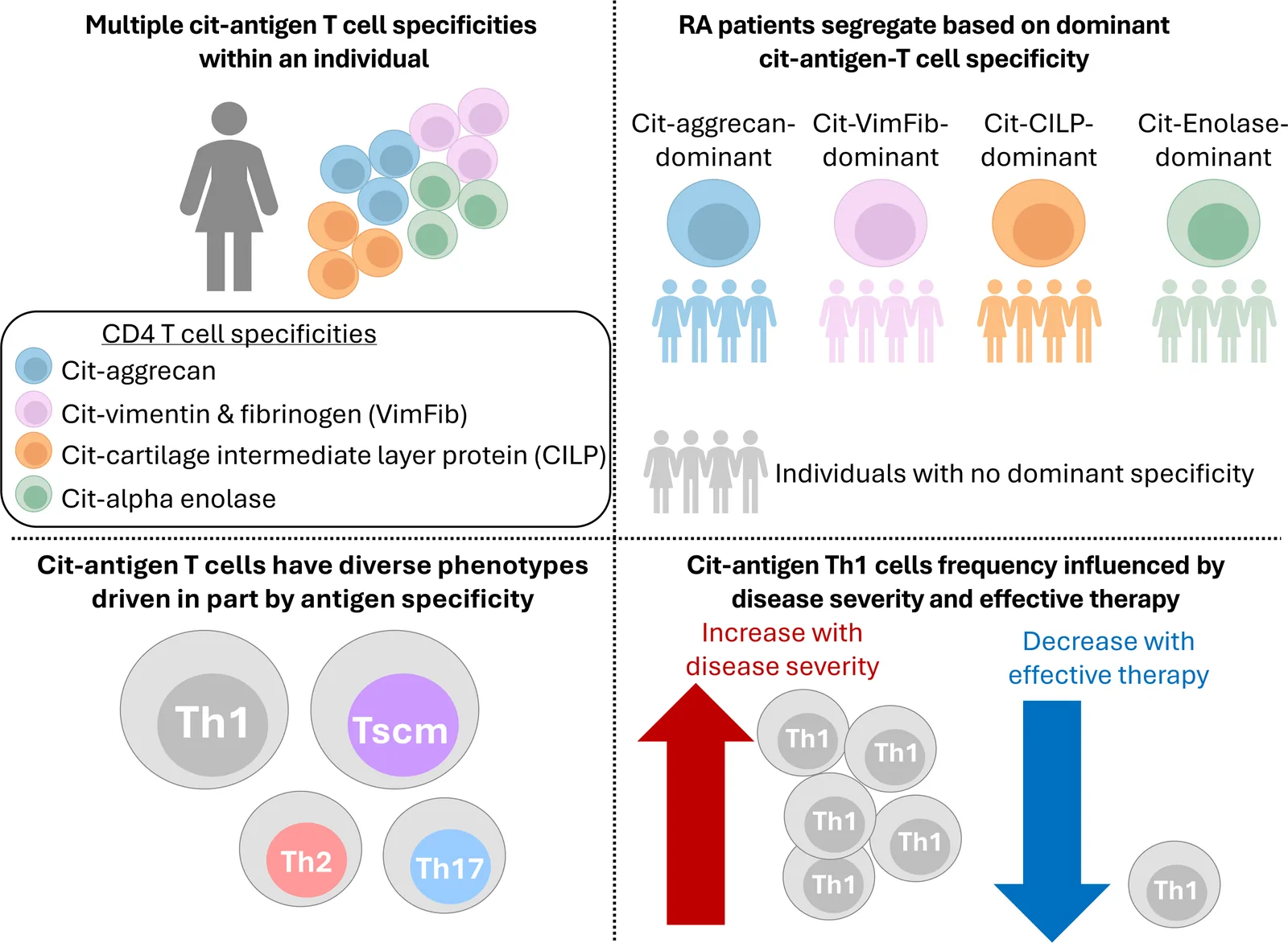

## Introduction

Rheumatoid arthritis (RA) is a debilitating chronic form of inflammatory arthritis that affects an estimated 23 million people worldwide (1). Current treatments include immunomodulatory drugs that lead to broad immunosuppression and require continuous treatment to control symptoms and joint destruction. Seropositive RA is characterized by the presence of rheumatoid factor and anti–citrullinated protein antibodies (ACPAs) and is strongly associated with HLA class II alleles referred to as the shared epitope. The role of immune recognition of citrullinated antigens (cit-antigens) in the development and ongoing pathology of RA is supported by the presence of ACPAs before diagnosis and at the onset of RA (2–4). Furthermore, immunization with ACPA target proteins found in the RA joint results in the development of arthritis in mice (5, 6).

CD4^+^ T cells are implicated in the pathogenesis of RA based on genetic association with HLA class II alleles (7), the presence of CD4^+^ T cells in the rheumatoid joint (8, 9), and the efficacy of T cell–directed therapies (10). Furthermore, ACPAs present in RA have a high level of somatic mutation, indicating T and B cell collaboration (11). In RA, the synovial lining of affected joints is infiltrated by CD4^+^ and CD8^+^ T cells (12, 13), and these have been shown to display an activated phenotype (14). Importantly, T cell receptor (TCR) repertoire analysis of samples from the blood and synovial tissue of seropositive RA patients revealed clonal expansion of T cells with disease-associated phenotypes, suggesting antigen-driven expansion (15). We have previously shown that T cells specific for cit-epitopes are increased in RA (16–18) and that their frequency is influenced by disease duration and therapy (16). Two studies have also reported the preferential accumulation of Th1-polarized cells within inflamed joints (13, 19). A variety of T cell subsets have implicated roles in the disease and similar T cell subsets are expanded in the peripheral blood of at-risk ACPA-positive individuals (20, 21). Despite these findings, technical challenges due to the rarity of cit-specific) T cells have limited our understanding of how cit-specific T cell phenotype and specificity can vary within and between individuals. To overcome these challenges, we and others have developed methods to simultaneously assess multiple antigen specificities ex vivo using HLA class II tetramers (22–24). Importantly, these methods allow us to address fundamental questions about the CD4^+^ T cell response in RA to known cit-autoantigens. These questions include whether there is a dominant cit-autoantigen that drives disease development and progression within an individual, whether the CD4^+^ T cell response to cit-autoantigens differs based on disease activity, whether there is a dominant autoreactive CD4^+^ T cell phenotype in RA, whether autoreactive CD4^+^ T cell phenotype is influenced by disease activity or therapy, and whether key baseline characteristics or longitudinal changes in the number and phenotype of cit-specific T cells correlate with treatment outcome. Answering these questions will provide invaluable insight into how therapies can effectively target the CD4^+^ T cell response based on the cellular phenotype in patients with RA.

In this current study, we combine multiplex tetramer staining with a specialized computational approach to directly interrogate cit-specific CD4^+^ T cells in a cross-sectional cohort of individuals with seropositive RA and HLA-matched healthy control (HC) participants. We found that cit-specific CD4^+^ T cells are increased in frequency in RA and target multiple autoantigens, within and across individuals. Furthermore, we observed a relationship between disease activity, the dominance of an autoantigen response, and frequency of Th1-like subsets. Finally, in a longitudinal cohort of individuals with active disease we observed changes in the numbers of cit-specific Th1 CD4^+^ T cells over time that correlated with treatment response.

## Results

### Applying HLA tetramers to study cit-antigen–specific T cells in HC and RA participants.

To enable broad characterization of T cell responses directed toward multiple cit-antigens, we applied a previously developed multiplex HLA class II tetramer staining approach (23, 25) to assess the frequency and surface phenotype of CD4^+^ T cells that bind HLA-DRB1*04:01 tetramers loaded with cit-peptides (referred to henceforth as cit-specific CD4^+^ T cells) within peripheral blood samples from a cross-sectional cohort of 64 seropositive RA and 27 HC participants, all of whom were DRB1*04:01 positive (characteristics summarized in Table 1). Our approach allowed highly reproducible detection of T cells that bind tetramers loaded with peptides from well-established synovial antigens (Supplemental Figure 1; supplemental material available online with this article; https://doi.org/10.1172/jci.insight.202159DS1). The tetramer panel included 11 previously defined cit-epitopes derived from 5 distinct synovial antigens: aggrecan (2 epitopes), vimentin (2 epitopes), fibrinogen (2 epitopes), cartilage intermediate layer protein (CILP) (2 epitopes), and α-enolase (3 epitopes); note that vimentin and fibrinogen were pooled (VimFib), so we had a total of 4 cit-antigen pools. We also included 2 influenza epitopes (CaHA 265-284 and TxHA 321-340) (23) for comparison to a foreign antigen. Each of these epitopes was previously shown to be DRB1*04:01 restricted and disease relevant (Supplemental Table 1). To draw inferences about T cell phenotype, we assessed the surface expression of chemokine receptors (CXCR3, CCR4, and CCR6), maturation markers (CD45RA and CCR7), and a long-term activation marker (CD38) (Supplemental Table 2).

Using a previously established limit of detection of 1 tetramer-positive cell per million CD4^+^ events (26), we were able to detect all 4 pools of cit-antigens in the majority of participants in both the RA and HC cohorts (Table 2 and Supplemental Figure 2A). We first asked whether the number of cit-antigen specificities detected differed between the RA and HC cohorts. The majority of participants in both cohorts had detectable levels of all 4 cit-antigen specificities and there were no significant differences between cohorts: 65.6% of the RA cohort versus 63.0% of the HC cohort (Supplemental Figure 2B). Likewise, similar percentages of the RA and HC cohorts had 3 detectable specificities (21.9% of the RA cohort versus 22.2% of the HC cohort), or 2 detectable specificities (12.5% of the RA cohort versus 11.1% of the HC cohort); a single healthy individual had 1 detectable specificity (Supplemental Figure 2B). We observed no significant differences in the frequency of cit-antigen–specific T cells based on sex, age, or disease duration (Supplemental Figure 3). Therefore, detectable numbers of cit-antigen specific T cells of multiple specificities are present in individuals with RA and HC, allowing us to ask questions about the frequency of these T cells in health and disease.

### Cit-antigen–specific T cells are increased in RA and associated with active disease.

**Figure 1:**
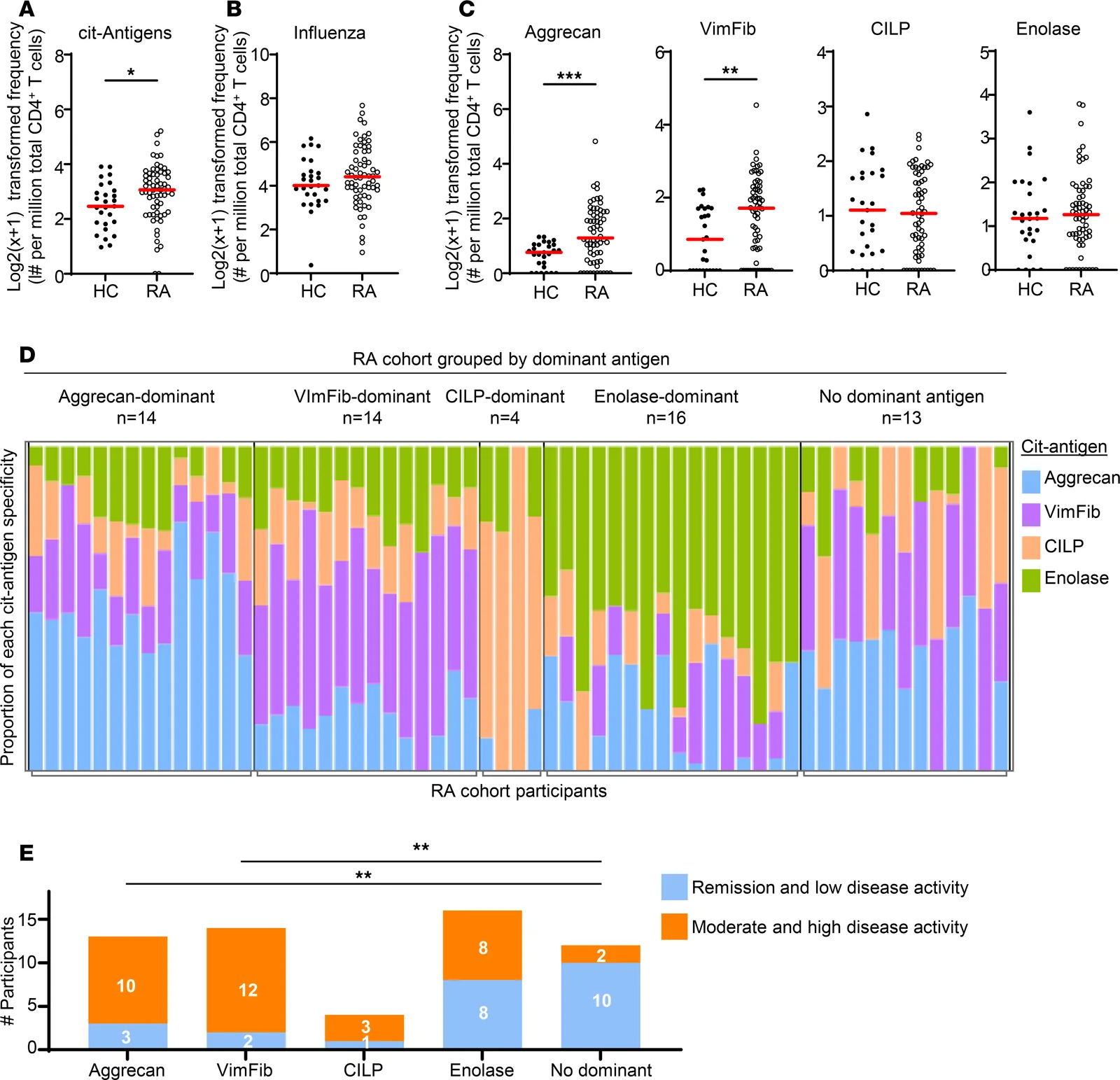
Cit-specific CD4^+^ T cells are increased in RA and exhibit antigenic focus in active disease. Ex vivo frequencies of non-naive T cells for (**A**) combined cit-antigens (*P* = 0.0148), (**B**) influenza antigen, and (**C**) cit-antigens individually (aggrecan *P* = 0.0004; VimFib *P* = 0.0078). For **A**–**C**, frequency (*y*-axis) was log_2_(*x* + 1) transformed; symbols represent individual participants (HC *n* = 27, RA *n* = 64) and horizontal bars show the median. *P ≤ 0.05, **P ≤ 0.01, ***P ≤ 0.001 by 2-tailed non-parametric Wilcoxon test. (**D**) Stacked bar plot where each column is an RA participant (*N* = 61) showing the proportion of each non-naive antigen frequency. The participants were grouped based on individual antigen dominance, where a *z* score was calculated for each antigen within each individual participant. A dominant antigen was defined as a *z* score of 1.0 or greater and those participants defined as “none” were undefined due to an all-antigens *z* score of less than 1.0; numbers within groups are participant counts. Note that 3 RA participants are not shown, as their non-naive frequencies were zero or total event counts were less than 8. (**E**) Bar graph summarizing the number RA participants with high or low disease activity (wRAPID3 < 2.0 are considered “low” and wRAPID3 > 2.0 are considered “active”) for individuals in each dominant antigen group (as depicted in **D**). Note that 2 of the participants shown in **D** did not have reportable wRAPID3 at time of draw; **P ≤ 0.01 by 2-way Fisher’s exact test against those participants grouped as “none.”

We compared the frequency of combined cit-antigen–specific CD4^+^ T cells in the RA and HC cohorts. Consistent with our previous study analyzing individual antigen specificities (16), the frequency of non-naive cit-specific CD4^+^ T cells was significantly increased in individuals with RA compared with HC participants (Figure 1A), whereas the frequency of influenza-specific CD4^+^ T cells did not differ between RA and HC (Figure 1B). CD4^+^ T cells specific for cit-aggrecan and cit-VimFib demonstrated the greatest increase in RA compared with HC (Figure 1C). Focusing on the RA cohort, we first asked whether the frequency of total RA antigen–specific T cells or the frequency of any individual specificity correlated with disease activity. When we modeled disease activity using the weighted RAPID3 (wRAPID3) score (27, 28) as a continuous variable, there was no significant correlation between T cell frequency and disease activity regardless of whether all cit-antigens were combined or examined individually (Supplemental Figure 4, A–C). Based on these analyses, we conclude there is no straightforward relationship between T cell frequency and wRAPID3 disease activity. Next we grouped individuals based on their dominant cit-antigen specificity and identified 5 distinct groups: individuals dominant for aggrecan (*n* = 14, 23%), VimFib (*n* = 14, 23%), CILP (*n* = 4, 7%), α-enolase (*n* = 16, 26%), and individuals with no dominant antigen (*n* = 13, 21%) (Figure 1D). To determine whether the dominant antigen specificity was associated with disease duration, we classified individuals with RA who had a defined cit-antigen focus as either short disease duration (less than 5 years) or a longer disease duration (more than 5 years). Grouping participants in this way (Supplemental Figure 5) revealed a possible bias towards aggrecan-dominant responses for individuals with a longer disease duration but that trend did not reach statistical significance (*P* = 0.21). Therefore, although we suspect that antigens such as aggrecan could become more dominant with longer duration (and other antigens such as CILP might become less dominant), our current data are not adequate to draw a firm conclusion. To determine whether having a distinct dominant antigen specificity is associated with higher disease activity, we performed a contingency analysis using the wRAPID3 score as a measure of disease activity. Notably, 77% of aggrecan-dominant individuals and 86% of VimFib-dominant individuals had active disease, whereas 83% of those without a dominant specificity had low RA disease activity (Figure 1E and Supplemental Figure 6). Furthermore, having aggrecan or VimFib as a dominant antigen was significantly associated with having active disease relative to having no dominant antigen (aggrecan *P* = 0.0048; VimFib *P* = 0.0011). Notably, a similar analysis was performed based on frequency of cit-specific T cells and no statistical difference in T cell frequency between the high and low disease activity groups was observed (Supplemental Figure 4, D–F).

### Cit-antigen–specific T cells in the blood of HC and RA participants have diverse phenotypes.

**Figure 2:**
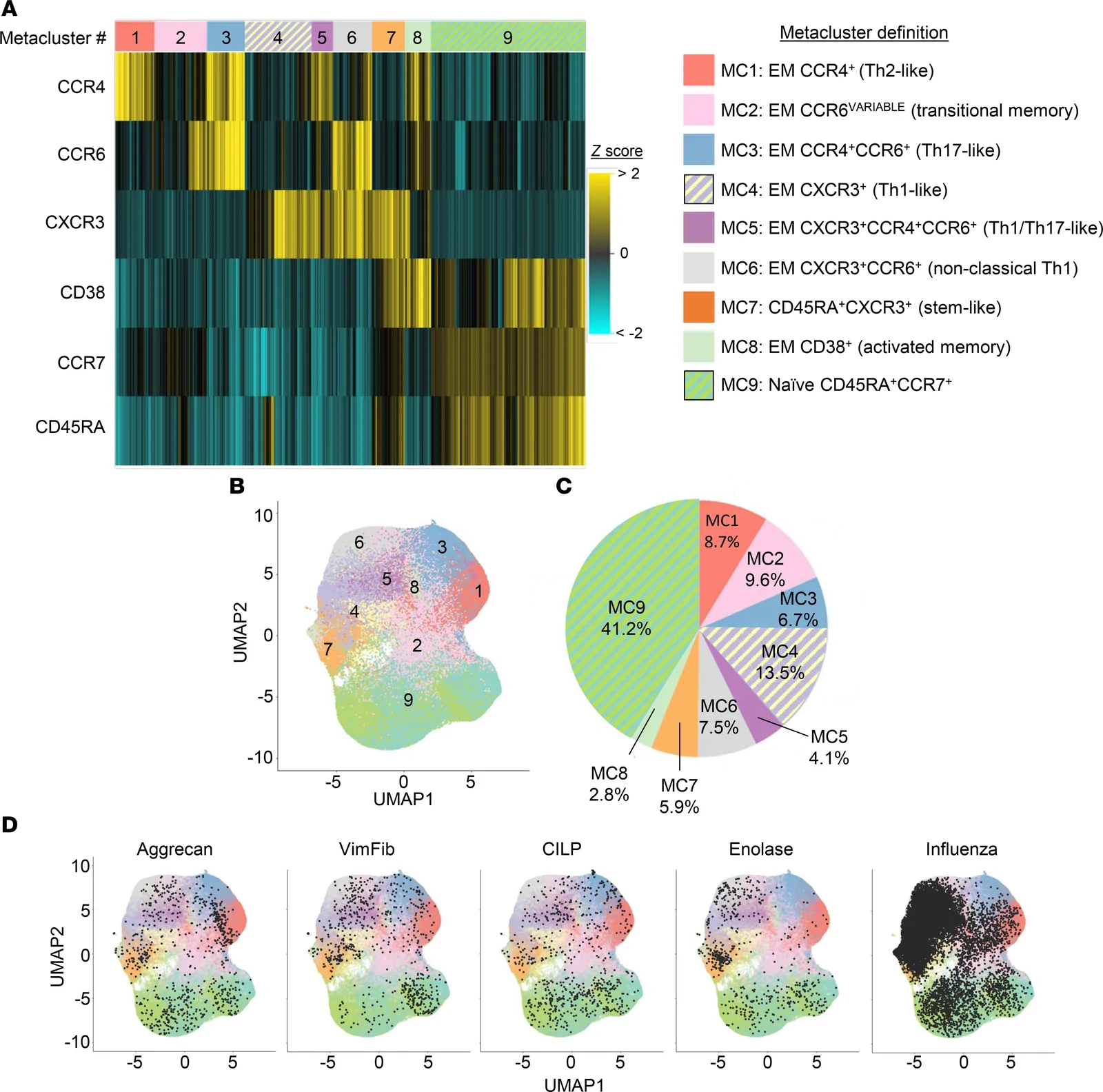
Defining the CD4^+^ T cell landscape using DISCOV-R reveals cit-specific T cell phenotypes. (**A**) Heatmap showing CD4^+^ T cell phenotypes from 64 RA and 27 HC participants, hierarchically clustered by expression of 6 phenotyping markers as a *z* score comparing mean cluster intensity to total CD4^+^ T cell intensity for each participant. The resulting dendrogram is sliced into 11 metaclusters (MCs), with the color bar across top indicating MC assignment. Note that boxes within the color bar are MCs that were combined based on similar marker overlap. (**B**) UMAP depicting bulk CD4^+^ T cell landscape and the locations of each assigned MC. (**C**) Pie chart depicting the mean frequency of each MC as a percentage of bulk CD4^+^ T for all participants (*N* = 91). (**D**) UMAPs displaying the occupancy of each antigen from all participants (black dots) overlayed on the CD4^+^ T cell landscape.

Next, we applied the robust computational approach distribution analysis across clusters of a parent population overlaid with a rare subpopulation (DISCOV-R; see Methods) to directly compare cell surface phenotypes based on antibody staining with an informative panel of cell surface markers. This tool is designed to compare T cell phenotypes within and across participants (without masking individual heterogeneity) and to perform an unbiased assessment of the phenotypic distribution of rare, tetramer-positive T cells (29). In brief, we first clustered total CD4^+^ T cells from each individual using Phenograph (30) and then performed hierarchical metaclustering to align clusters between individuals, thereby generating a common CD4^+^ T cell landscape across all participants (Figure 2, A and B). Using this approach, we determined that the overall CD4^+^ T cell landscape consisted of 9 metaclusters (MCs) that were assigned the following phenotypes: effector memory (EM) CCR4^+^ (MC1, Th2-like), EM CCR6^variable^ (MC2, transitional memory), EM CCR4^+^CCR6^+^ (MC3, Th17-like), EM CXCR3^+^ (MC4, Th1-like), EM CXCR3^+^CCR4^+^CCR6^+^ (MC5, Th1/Th17-like), EM CXCR3^+^CCR6^+^ (MC6, non-classical Th1), CD45RA^+^CXCR3^+^ (MC7, stem-like), EM CD38^+^ (MC8, activated memory), and Naive CD45RA^+^CCR7^+^ (MC9, naive). Naive cells were the most abundant MC phenotype (41.2%). The remaining non-naive phenotypes ranged from 2.8% to 13.5% of the total CD4^+^ T cell landscape (Figure 2C). To verify the functional accuracy of these MC assignments, we characterized IFN-γ, IL-4, and IL-17 expression by T cells with characteristic surface markers for each MC (Supplemental Table 3) and observed cytokine levels that supported our designations (Supplemental Figure 7). We next overlayed tetramer-positive cells onto the DISCOV-R–defined CD4^+^ T cell landscape (Figure 2D), enabling a contextualized assessment of the surface phenotypes of cit-antigen–specific CD4^+^ T cells. Tetramer-labeled T cells from all participants with at least 8 tetramer events were included in this analysis (Supplemental Table 4). Notably, we observed tetramer-positive cells distributed across the MCs.

### Distinct phenotypes are enriched among cit-antigen–specific T cells.

**Figure 3:**
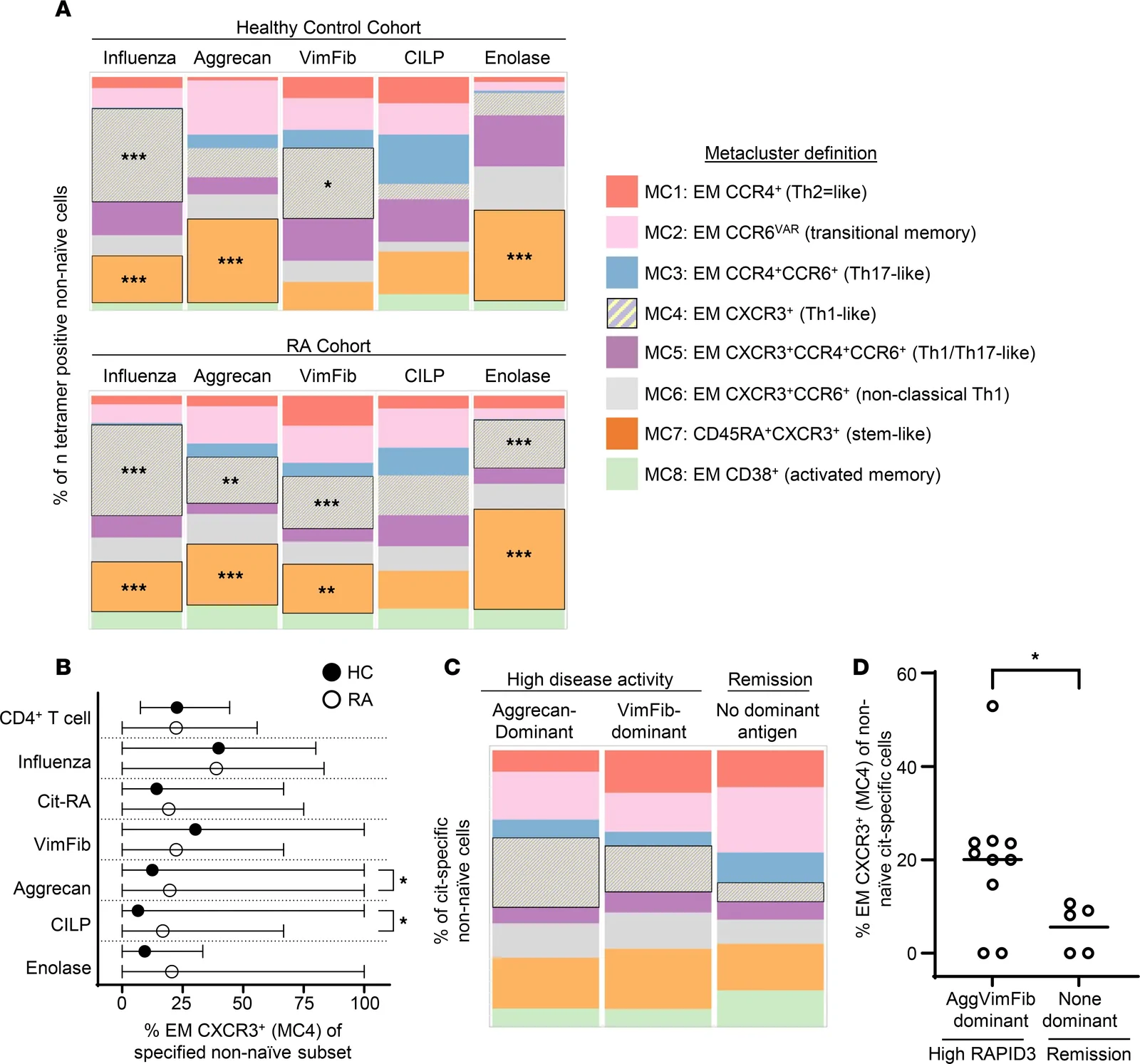
Among diverse cit-antigen–specific T cell phenotypes, distinct metaclusters are increased in active RA. (**A**) Stacked bar graph of non-naive phenotype frequencies of each cit-antigen– and influenza-specific CD4^+^ T cells for HC and RA participants, where prominent metaclusters (MCs) within each antigen were identified by first testing for unequal variance by comparing the group means of the absolute deviations from the median (ADM) to the overall mean ADM, then MCs exceeding an upper limit (α = 0.1) were boxed and tested for statistical significance by comparing to the unboxed MCs using samples with at least 8 parent events — influenza (*n* = 88), aggrecan (*n* = 55), VimFib (*n* = 47), CILP (*n* = 52), and α-enolase (*n* = 54) (Supplemental Table 4). (**B**) Total, influenza-specific, and cit-antigen–specific percentages of MC4 (EM CXCR3^+^) based on disease status. HC, closed circles; RA, open circles. Kruskal-Wallis test with multiple comparisons: aggrecan *P* = 0.0270 and CILP *P* = 0.0214. (**C**) Stacked bar graph of cit-RA CD4^+^ T cell phenotype distribution for those RA participants with high disease activity (wRAPID3 > 4.0) defined by aggrecan (*n* = 4) and VimFib (*n* = 6) dominance and participants near remission (wRAPID3 < 1.0) no dominance (*n* = 5); boxed are MC4 (EM CXCR3^+^) for each group. (**D**) Percentage MC4 (EM CXCR3^+^) of non-naive cell subset for individuals with high disease activity who are dominant for either aggrecan or VimFib compared with individuals in remission who have no dominant antigen specificity. *P ≤ 0.05, **P ≤ 0.01, ***P ≤ 0.001 by non-parametric Wilcoxon test.

Focusing on the non-naive MCs, we see tetramer-positive cells fall into all MCs (Figure 3A). As expected, influenza-specific CD4^+^ T cells were significantly enriched within MC4 (EM CXCR3^+^, Th1-like) and MC7 (CD45RA^+^CXCR3^+^, stem-like) in both HC and RA. In HC, there was enrichment for MC7 (CD45RA^+^CXCR3^+^, stem-like) specifically among aggrecan- and α-enolase–specific T cells. Within the RA cohort, there was significant enrichment not only in MC7 but also MC4 for all cit-antigen specificities except CILP, establishing MC4 as a uniquely important cluster for individuals with RA. Notably, the percentage of MC4 cells (EM CXCR3^+^, Th1-like) showed substantial variation among the different cit-antigen specificities). Given this variation, we asked whether the frequency of Th1-like cells within MC4 differed between RA and HC (Figure 3B) and observed that the frequency of Th1-like cit-aggrecan– and cit-CILP–specific T cells was significantly higher in individuals with RA (*P* = 0.027 and *P* = 0.0214, respectively). To ask whether the MC4 phenotype is increased in active disease, we compared the percentage of cells with this phenotype in aggrecan- and VimFib-dominant individuals with high disease activity (wRAPID3 > 4.0) to individuals with no dominant antigen and remission disease activity (wRAPID3 < 1.0). Notably, the high disease activity aggrecan- and VimFib-dominant groups had an elevated percentage of T cells found within MC4, as compared with the remissive disease/no-dominant-antigen group (Figure 3, C and D); this was not observed for influenza-specific CD4^+^ T cells (Supplemental Figure 8A). As an alternative analysis method, we applied a linear mixed modeling approach, modeling antigen-specific cell frequencies by cluster with donor as a random variable. This linear mixed model revealed that VimFib-specific T cells predominantly occupied MC4 in individuals with high disease activity (Supplemental Figure 8B). In a subset of individuals, the MC7 phenotype clusters appeared to predominate for other specificities (for α-enolase and aggrecan) but that enrichment did not reach statistical significance (Supplemental Figure 8B). Together, these findings suggest that cit-antigen–specific T cells take on a wide range of phenotypes, but certain cell states (Th1-like and stem-like) predominate and that the proportion of Th1-like cells may be increased in active disease.

### Cit-antigen–specific T cell frequency and phenotype in a longitudinal cohort.

Motivated by the associations we observed in the cross-sectional RA cohort, we sought to examine dynamic changes in the frequency and attributes of T cells specific for these cit-antigens in a longitudinal sample set (Supplemental Table 5). Like the cross-sectional sample set, we used multiplex tetramer and antibody staining panels to label and enumerate cit-specific T cells and characterize their cell surface phenotype (Supplemental Tables 6 and 7). Using DISCOV-R, we defined a longitudinal CD4^+^ T cell landscape that consisted of 10 MCs (Supplemental Figure 9). Combining closely related MCs based on distance and overlap within the UMAP space yielded 6 refined longitudinal MCs (LMCs), which were assigned the following phenotypes: CD45RA^+^CXCR3^+^ (LMC1, stem-like), EM CXCR3^+^ (LMC2, Th1-like), CXCR3^dim^CCR4^neg^CCR6^var^ (LMC3, nonclassical Th1), CCR4/CCR6 (LMC4, Th17-like), CXCR3^neg^CCR4^var^CCR6^var^ (LMC5, Th1/Th17-like), and Naive CD45RA^+^CCR7^+^ (LMC6, naive). So, 6 of the 10 LMCs corresponded to 6 MCs found in the cross-sectional CD4^+^ T cell landscape, including the key MC4 that varied with disease status, therapy, and disease activity. The longitudinal landscape did not have an LMC corresponding to MC1 (Th2-like cells), MC2 (transitional memory), and MC8 (activated memory). To validate our findings from the cross-sectional cohort, we analyzed baseline data from the longitudinal cohort and found that the most frequent antigen specificities and phenotype clusters revealed through DISCOV-R were similar for the 2 cohorts (Supplemental Figure 10). Notably, the aggrecan- or VimFib-dominant T cell responses were associated with active disease, as observed in the cross-sectional cohort (Supplemental Figure 10, C and D).

### Changes in cit-antigen–specific T cell frequency and phenotype correlate with treatment response.

**Figure 4:**
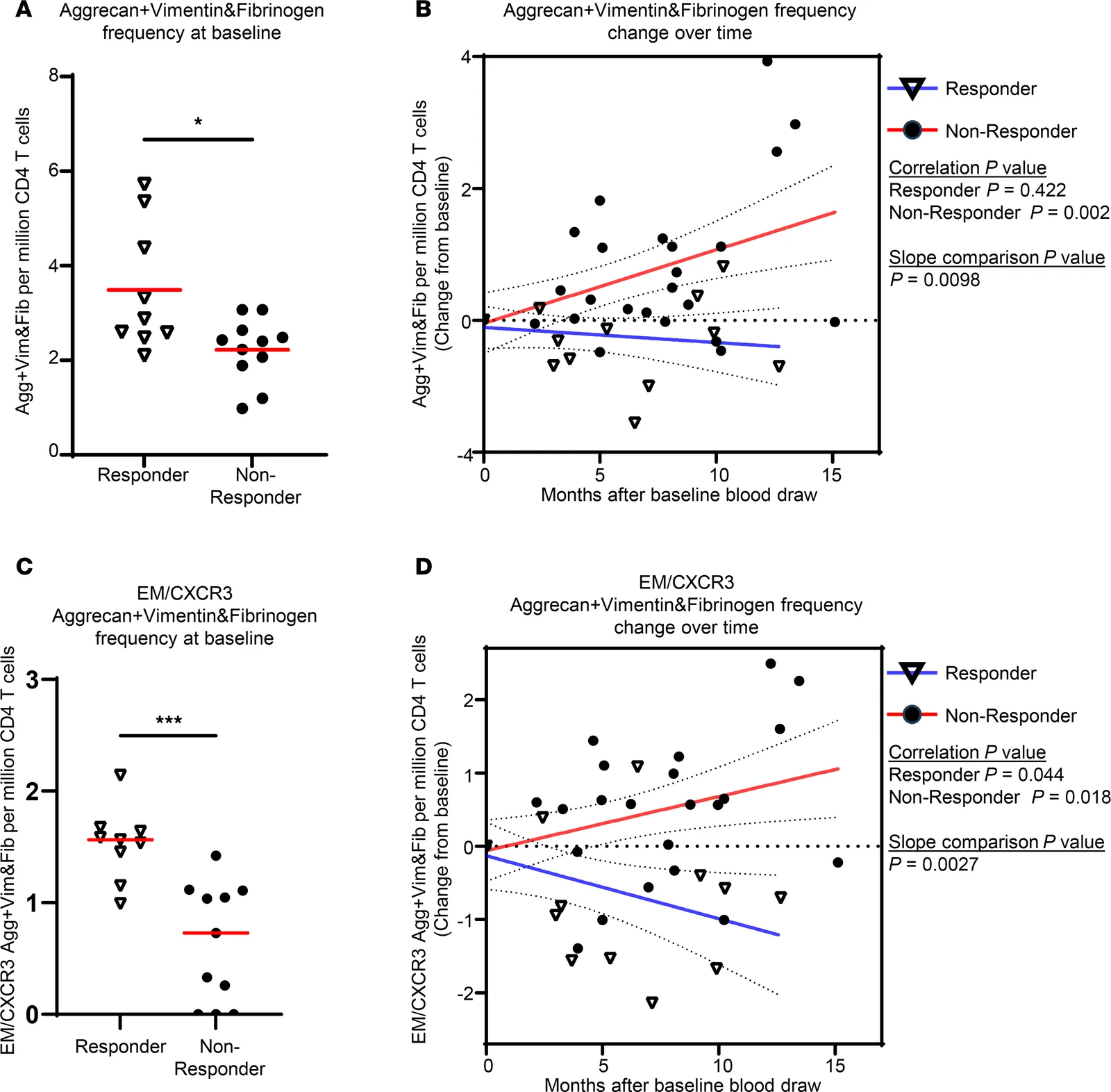
The frequency of cit-specific Th1-like cells is decreased individuals who respond to therapy. The frequencies of cit-aggrecan– and cit-VimFib–specific T cells were combined from RA participants who had blood draws before and after starting a new therapy. At first draw, all individuals had active disease based on a weighted wRAPID3 score of 2.3 or higher. These individuals were subsequently defined as Responder or Non-Responders based on their weighted RAPID3 at second draw (Responder wRAPID3 < 2.0, Non-Responder wRAPID3 ≥ 2.3; see Supplemental Figure 11). (**A**) Frequency of cit-Agg– and cit-VimFib–specific CD4^+^ T cells in Responders (*n* = 9) and Non-Responders (*n* = 11); 2-tailed non-parametric Wilcoxon test. (**B**) The frequency of cit-Agg– and cit-VimFib–specific CD4^+^ T cells in Responders (*n* = 20) and Non-Responders (*n* = 33) was normalized to initial draw and then plotted against the number of months thereafter; simple linear regression model, 95% confidence intervals, and 2-tailed Spearman’s correlation where non-responders *P* = 0.0020 and slope compared to responders calculated as *P* = 0.0098. (**C**) The baseline frequency of cit-antigen–reactive Th1-like (EM CXCR3^+^) cells in responders (*N* = 9) were compared to non-responders (*N* = 11); 2-tailed non-parametric Wilcoxon test. (**D**) The frequency of cit-antigen–reactive Th1-like (EM CXCR3^+^) cells in Responders (*n* = 20) and Non-Responders (*n* = 33) was normalized to initial draw and then correlated with months after baseline draw prior to new therapy; non-parametric Spearman correlation was used to determine correlation to time (responder *P* = 0.044, non-responder *P* = 0.018) and simple linear regression was used to determine slope differences between the 2 groups (*P* = 0.0027). For **C** and **D**, data points are those samples where parent event count is at least 8. Graphs **A**–**D** are log_2_(*x* +1) transformed. *P ≤ 0.05, ***P ≤ 0.001.

The longitudinal cohort included 20 DRB1*04:01-positive individuals with seropositive RA who had high disease activity (defined by wRAPID3 score ≥ 2.3) and who were transitioning to a new therapy (Table 3). This enabled us to examine dynamic changes in the frequency and attributes of cit-antigen–specific T cells based on response to therapy. Participants were classified as responders if their wRAPID3 score decreased to below 2.3 after transition to new therapy or non-responders if they maintained a wRAPID3 scores of 2.3 or higher (Supplemental Figure 11). This allowed us to compare cit-specific CD4^+^ T cell frequencies and phenotypes in responders versus non-responders, focusing on cit-aggrecan– and cit-VimFib–specific cells combined (cit-AggVimFib). At baseline, cit-AggVimFib–specific T cell frequencies were higher in responders compared with non-responders (Figure 4A). Notably, cit-AggVimFib–specific T cell frequencies increased over time in non-responders but did not change in responders (Figure 4B). In contrast, influenza-specific T cells were not influenced by changes in disease activity (Supplemental Figure 12A) but did increase over time among RA therapy responders (Supplemental Figure 12B). These data show that lack of treatment response is accompanied by an increase in cit-antigen–specific T cell number, whereas effective therapy may control T cell expansion. We next investigated longitudinal changes in cit-antigen–specific T cell phenotype in non-responders versus responders, with a focus on the LMC2 cluster that resembled the Th1-like cluster that was important in the cross-sectional cohort. At baseline, the frequency of cit-AggVimFib–specific Th1-like cells was elevated among responders compared with non-responders (Figure 4C); similar elevation was not observed for influenza-specific Th1-like cells (Supplemental Figure 12C). Furthermore, in samples taken after introduction of a new therapy (Supplemental Figure 11), the frequency of these cit-AggVimFib–specific Th1-like cells decreased among responders (Supplemental Figure 13A). Moreover, there was a decrease in the normalized frequency of Th1-like cit-antigen–specific T cells for responders but an increase for non-responders (Figure 4D and Supplemental Figure 13A). There were no corresponding changes for Th1-like influenza-specific T cells in these same samples, suggesting differential modulation of cit-specific responses only (Supplemental Figure 12D and Supplemental Figure 13B).

## Discussion

Our study focused on characterizing cit-specific CD4^+^ T cell responses for individuals with seropositive RA, which is justified, given that the presence of cit-specific autoantibodies mark an at-risk population that is likely to develop RA (31) and ACPAs are recognized as a strong clinical predictor of radiologic disease progression (32–34). Prior studies (our own and the work of others) have investigated CD4^+^ T cell responses against several cit-antigens, including aggrecan, vimentin, fibrinogen, CILP, and α-enolase (35–40), defining these as relevant T cell targets in RA. Likewise, prior studies have shown that T cells specific to cit-peptides derived from those antigens are present in patients with RA, exhibit an expanded memory phenotype, and can have an inflammatory Th1 phenotype (16, 17, 24, 41, 42). Notably, antigens such as vimentin are citrullinated within neutrophilic NETs and can be presented to T cells by synovial fibroblasts, a mechanism that may sustain pathogenic autoimmunity in affected joints (43). Each of these published studies focused on a single cit-antigen of interest. Recently developed methods allow simultaneous interrogation of multiple antigen specificities in a single sample (22–24), enabling more comprehensive study of cit-antigen–specific CD4^+^ T cell responses in individuals with RA. Using a robust multi-color tetramer staining approach, we assessed the frequency of cit-antigen–specific T cells in the peripheral blood of individuals in cross-sectional RA and HC cohorts. All cit-antigen specificities were detectable in peripheral blood and, as expected, we observed higher overall T cell frequencies in RA than HC. Our multi-color, multi-antigen approach revealed that individuals with RA may be differentiated by distinct “dominant” antigens. In particular, aggrecan and VimFib dominance was enriched among individuals with active disease. To examine T cell phenotypes, we applied a computational analysis strategy designed to minimize biases and facilitate comparisons of complex T cell phenotypes (29) to define the landscape of CD4^+^ T cell responses. The landscape observed for the cross-sectional cohort consisted of 9 distinct MCs with features that were consistent with previously described T cell subsets. Cit-specific T cells were distributed across all MCs but were enriched within Th1-like (MC4) and stem-like (MC7) MCs. Active RA was associated with having an aggrecan- or VimFib-focused T cell response and having a higher proportion of cit-specific T cells within the Th1-like MC4.

To bolster findings obtained for the cross-sectional cohort, we investigated the number and attributes of cit-antigen–reactive T cells in baseline samples from the longitudinal cohort. For both cohorts, the most frequent antigen specificities and the range of phenotype clusters identified by DISCOV-R were highly similar. Parallel analysis of both cohorts affirmed the predominance of aggrecan- or VimFib-dominant T cell responses in active disease and the relevance of Th1-like (MC4 and LMC2) cit-reactive T cell phenotypes. Prior studies established the importance of IL-17–producing and Th1-polarized cells for driving disease (44), but also support the relevance of other T cell subsets, including peripheral helper T (Tph) cells (defined as having a CXCR5^–^PD1^+^ surface phenotype), CD28^–^ T cells (seen within the synovium of RA patients), and a CD27^–^HLA-DR^+^ CD4^+^ T cell population (reported to be present in the joints of seropositive RA patients) (45–47). Due to the markers used in our studies we were unable to assess these cell populations, which would be of interest in future studies.

Our study does have limitations. Regrettably, for technical reasons our staining panel was limited to 6 phenotypic surface markers, forcing us to make difficult decisions about which to include. Future studies should include additional markers and apply single-cell transcriptional approaches to shed even greater light on the breadth of T cell phenotypes that are important in RA. In addition, we were unable to include recently described RA-associated T cell specificities, in part based on the limitation of the number of tetramers that we could incorporate at the time of this study. Furthermore, the methods we applied did not allow interrogation of CD8^+^ T cells, which are prevalent in inflamed joints, exhibit important disease associated phenotypes, and have recently been shown to respond to synovial cit-antigens (13, 15, 48). In addition, individuals in our longitudinal cohort were predominantly treated with TNF inhibitors, so we were unable to ask about differences based on therapy type. Furthermore, our work was limited to the study of peripheral blood. There could be a disconnect between the T cell phenotypes present in peripheral blood and T cell phenotypes present in inflamed joints, such that T cell phenotypes that are important in tissue are not well represented in the periphery. However, a substantial proportion of T cells in synovial fluid were reported to be CXCR3 positive, supporting the relevance of the Th1-like cluster that stood out in our cross-sectional and longitudinal data sets (49).

Despite these limitations, some important insights emerge from our study’s observations. Our results indicate that there is no single cit-antigen T cell specificity that is predominant across all individuals with RA, but in most individuals a single antigen specificity predominated, and certain dominant specificities (aggrecan, vimentin, and fibrinogen) were associated with active disease. This could suggest a disease process in which failed tolerance toward initiating antigens is followed by an expansion of the T cell responses toward new cit-antigens that serve to mark active disease. This would mirror the reported characteristics of ACPAs, for which there is an expansion of targets leading to RA development (4, 11, 50). Cit-specific T cells had diverse phenotypes, but 2 clusters — a Th1-like cluster and a stem-like cluster — were most enriched among cit-specific T cells. The Th1-like cluster could be an important biomarker of disease activity and treatment response. The stem-like cluster did not exhibit meaningful variation with disease activity or therapy but could represent an important reservoir that can expand into pathogenic effector cells at sites of inflammation, as has been observed in autoimmune vasculitis and ulcerative colitis (51, 52). As such, additional studies aimed at understanding both T cell subsets are warranted. Ideally, new studies would include a broader panel of surface markers (to include pathogenic subsets such as Tph cells) and transcriptomic analysis to achieve a deeper understanding of the dynamics, interplay, and heterogeneity of cit-specific T cell phenotypes.

In conclusion, our study shows that cit-specific CD4^+^ T cells have broad specificities and dynamic functional immunophenotypes in established RA. The expansion of CD4^+^ T cell responses toward multiple cit-antigens across individuals argues against a single antigenic driver of disease, instead implying a broader failure of tolerance, implicating autoreactive B cells, T cells, and the innate response. However, the finding that dominant antigenic responses can differentiate individuals may suggest discrete factors that promote the expansion of certain specificities. The changes in cit-specific frequency with treatment response does suggest that T cells are markers of disease activity as a reflection of their role in pathogenesis or as an indicator of joint-specific inflammation. Due to the broad array of specificities that comprise the cit-specific CD4^+^ T cell response, targeting a single epitope or antigen may not be sufficient to achieve immune tolerance. In this study, we examined individuals with established RA and found T cells reactive to aggrecan and VimFib to be dominant. This contrasts with our previous observation that CILP-specific T cells dominate in at-risk individuals sampled prior to development of RA (53), which suggests plasticity in the antigen specificity of T cell responses in individuals with RA over time. We observed a distinct Th1-like state whose depletion is associated with favorable treatment outcomes and expands in treatment non-responders. This suggests an ongoing remodeling of the autoimmune response during established disease, likely due to persistent inflammation, which can be interrupted with current therapies to achieve clinical benefit. Additional longitudinal profiling of cit-specific CD4^+^ T cells is likely to provide new mechanistic understanding of the T cell subsets that promote disease progression and flares and a means of assessing the immunologic impact of effective therapy. This in turn may suggest new therapies designed to target these distinct T cell subsets.

## Methods

### Sex as a biological variable.

Our study included both male and female participants (biological sex as reported at enrollment). The recruited population was biased towards females, reflecting the female bias of RA disease. Sex was considered as a biological variable and data for males and females were pooled in all cases where sex-based differences were not evident.

### Study participants.

All participants had at least one copy of the HLA-DRB1*04:01 allele. The cross-sectional cohort consisted of 64 seropositive RA participants and 27 HC participants (Table 1). The longitudinal cohort consisted of 37 seropositive RA participants (Table 3). The RA participants carried a diagnosis of RA based on the 2010 American College of Rheumatology criteria, were positive for ACPAs, and recruited from the Virginia Mason Medical Center and the VA Puget Sound Health Care System. HC participants had no first-degree relatives with autoimmune disease and were recruited through the Benaroya Research Institute Registry and Repository. All assays were run and analyzed in a blinded manner. Disease activity for each individual was determined using the wRAPID3 score (27, 28). Low disease activity was defined as wRAPID3 0–2.0 (remission + low activity), whereas high disease activity was defined as wRAPID3 2.1–10.0 (moderate + high activity).

### Isolation and cryopreservation of PBMCs.

PBMCs were isolated from heparinized blood by centrifugation over Ficoll-Hypaque gradients, and frozen in liquid nitrogen in 10% DMSO and 90% heat-inactivated FBS. Cryopreserved PBMCs were thawed in a 37°C water bath and thawing medium (RPMI-1640 supplemented with 10% FBS) plus 0.001% benzonase nuclease (Sigma-Aldrich) was added dropwise. Cells were pelleted and resuspended in complete medium (RPMI-1640 supplemented with 2 mM L-glutamine, 100 U/mL penicillin, 100 μg/mL streptomycin, 10 mM HEPES plus 10% commercial human pooled serum).

### HLA class II tetramer production.

Recombinant HLA-DRB1*04:01 protein was produced by the Benaroya Research Institute Tetramer Core, as previously described (54). Soluble HLA-DRB1*04:01 monomer was purified from insect cell culture supernatants and biotinylated at a sequence-specific site using biotin ligase (Avidity) prior to dialysis into phosphate storage buffer. The biotinylated monomer was loaded with 0.2 mg/mL of peptide by incubating at 37°C for 72 hours in the presence of 2.5 mg/mL *n*-octyl-β-D-glucopyranoside and 1 mM Pefabloc SC (Sigma-Aldrich). Monomers loaded with cit-peptides (synthesized by Sigma-Aldrich) known to be HLA-DRB1*04:01–restricted epitopes were conjugated into tetramers using streptavidin (Invitrogen) labeled with various fluorescent labels (Supplemental Tables 1 and 6) for 6–18 hours at room temperature at a molar ratio of 8:1.

### Ex vivo detection of cit-antigen– and influenza-specific T cells.

Ex vivo tetramer staining and enrichment was accomplished using previously published protocols (23, 24). A total of 20–30 million PBMCs were thawed, resuspended in 200 μL of T cell culture medium, and stained with labeled tetramers at room temperature for 90 minutes. Cells were washed and incubated with anti-PE, anti-APC, and anti-Myc magnetic beads (Miltenyi Biotec) at 4°C for 20 minutes, washed again, and a 1/100th fraction was saved for antibody staining (“Pre”). The other fraction was passed through a MS magnetic column (Miltenyi Biotec). Bound, PE-, PE-CY5–, PE-CF–, APC-, or BV421-labeled cells were flushed and collected. Both enriched (“Post”) and non-enriched (“Pre”) fractions were labeled with Sytox green– and fluor-conjugated antibodies (Supplemental Tables 2 and 7). Samples were collected on a BD FACS LSR Fortessa and analyzed using FlowJo v10 (both from Waters Biosciences) (gating strategy shown in Supplemental Figure 1). The frequency of tetramer^+^ CD4^+^ T cells was calculated as: F = (1,000,000 × tetramer^+^ events from enriched sample)/(100 × number of CD4^+^ cells from the non-enriched fraction).

### DISCOV-R computational analyses.

DISCOV-R analyses were performed as described previously (29). In brief, FCS files were analyzed using custom R scripts (https://github.com/BenaroyaResearch/Buckner_Linsley_Cit-specific) based on the flowCore (55), Rtsne (56–58), and cytofkit (30, 59–61) packages. Tetramer-positive events were concatenated to total CD4^+^ T cell FCS files for each sample prior to arcsinh transformation using the following parameters: a = 0, b = 1/150. Subsequently, t-SNE analysis and Rphenograph clustering (Individual Clusters) were performed for each sample using 6 phenotypic markers: CD45RA, CD38, CCR4, CCR6, CXCR3, and CCR7. Cluster alignment of Individual Clusters with greater than 1% frequency was performed across individuals by hierarchically metaclustering the *z*-score values of each cluster’s marker expression compared to each participant’s total CD4^+^ T cells, with Euclidean distance and Ward’s method used to assess phenotypic similarity; heatmaps were generated in R using the ComplexHeatmap package (62). The dendrogram was cut into segments using cutree from the R stats package (63), and the resulting cluster assignments (MCs) were applied to the individual samples. Total CD4^+^ and tetramer-positive T cells were counted for each aligned cluster in each participant, and their frequency was calculated.

### Antibody staining of CD4^+^ T cell populations defined by DISCOV-R.

To further characterize the computationally defined CD4^+^ T cell populations, PBMCs were stained for additional surface markers and cytokines with fluor-conjugated antibodies while maintaining the base antibody panel used for tetramer analysis (Supplemental Table 3). Briefly, cells were activated with PMA/ionomycin (BioLegend) or DMSO along with Brefeldin-A (BioLegend) treatment for 5 hours at 37°C. Cells were then stained with Zombie Green viability dye (BioLegend), stained with the surface marker base panel, then fixed, permeabilized, and stained using a fixation permeabilization kit (Thermo Fisher Scientific). All samples were collected on a BD FACS Fortessa and analyzed with FlowJO v10. Cytokine frequency was calculated by subtracting activated minus DMSO conditions for each sample.

### Statistics.

All statistical analyses were conducted using GraphPad Prism version 10.0 and JMP version 18.1. Tests were 2-tailed unless otherwise specified. For pairwise comparisons of non-normally distributed data, the nonparametric Wilcoxon rank-sum test was used. The Benjamini-Hochberg method was used to adjust the significance level (α = 0.05) when testing multiple hypotheses simultaneously. One-way ANOVA followed by Dunnett’s method for multiple comparisons to assess normally distributed data in multi-group comparisons such as those involving disease activity groups and antigen specificity. Fisher’s exact test was used to assess categorical variables, such as the relationship between disease activity groups and antigen specificity. The nonparametric Spearman correlation was used to evaluate monotonic relationships between variables, such as associations between response scores and clinical measurements. *P* values associated with Spearman correlation statistics tested the null hypotheses that the true correlation is zero.

### Study approval.

The studies described in this manuscript were conducted under Institutional Review Board–approved protocols at the Benaroya Research Institute (IRB07109) and the VA Puget Sound Health Care System (MIRB 00755). All participants gave written informed consent in accordance with the Declaration of Helsinki using consent forms approved under these protocols.

### Data availability.

A Supporting Data Values file has been provided with the manuscript and supplemental materials. The source code is available in GitHub (https://github.com/BenaroyaResearch/Buckner_Linsley_Cit-specific). Cytometric data are available in ImmPort (https://www.immport.org/shared/home; accession SDY3698).

## Author contributions

EAJ and JHB conceptualized and designed the study. JC, BN, JHB, SEP, and HB were responsible for participant selection and clinical data collection. HU and EAJ designed the staining panel. CR performed the T cell studies. VSM, HAD, and PSL performed statistical modeling and analysis. CR, EAJ, AMH, and JHB wrote the manuscript with assistance from all co-authors. JHB obtained funding and was responsible for the entire project.

## Conflict of interest

JHB is a scientific co-founder and Scientific Advisory Board member of GentiBio, consultant for Bristol Myers Squibb and Hotspot Therapeutics, and has past and current research projects sponsored by GentiBio, Amgen, Bristol Myers Squibb, Janssen, Novo Nordisk, and Pfizer. She is a member of the Type 1 Diabetes TrialNet Study Group, a partner of the Allen Institute for Immunology, and a member of the Scientific Advisory Boards for the La Jolla Institute for Allergy and Immunology and BMS Immunology. EAJ has received research support from Indupro and Nipuna Therapeutics.

## Funding support

- US Department of Defense grant W81XWH-15-1-0003 (to JHB). All opinions, interpretations, conclusions, and recommendations are those of the authors and are not necessarily endorsed by the US Department of Defense.
- Knut and Alice Wallenberg Foundation (to HU).

## Supplementary Material

Supplemental data

Supporting data values

## Figures and Tables

**Table 1:**
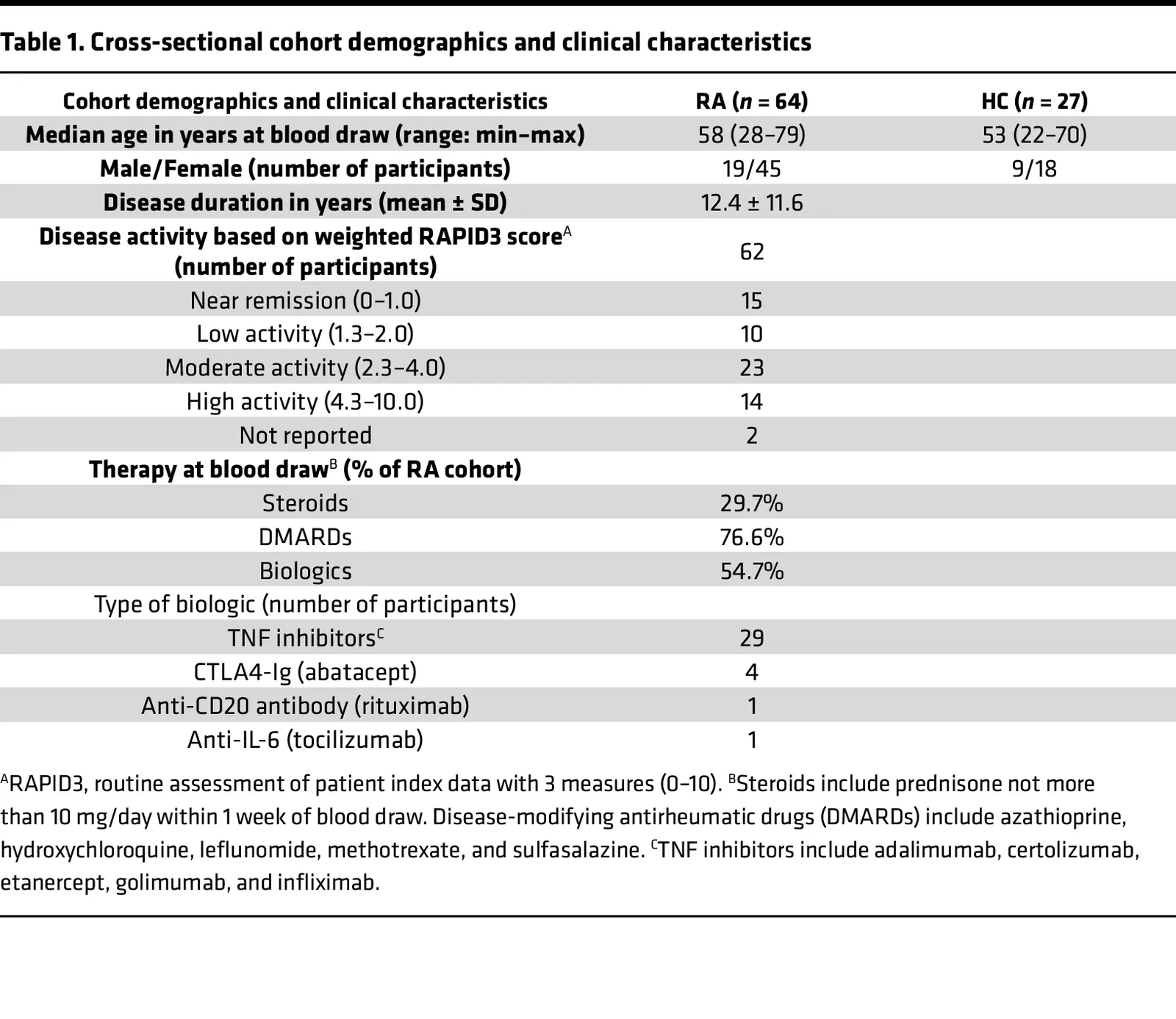
Cross-sectional cohort demographics and clinical characteristics

**Table 2:**
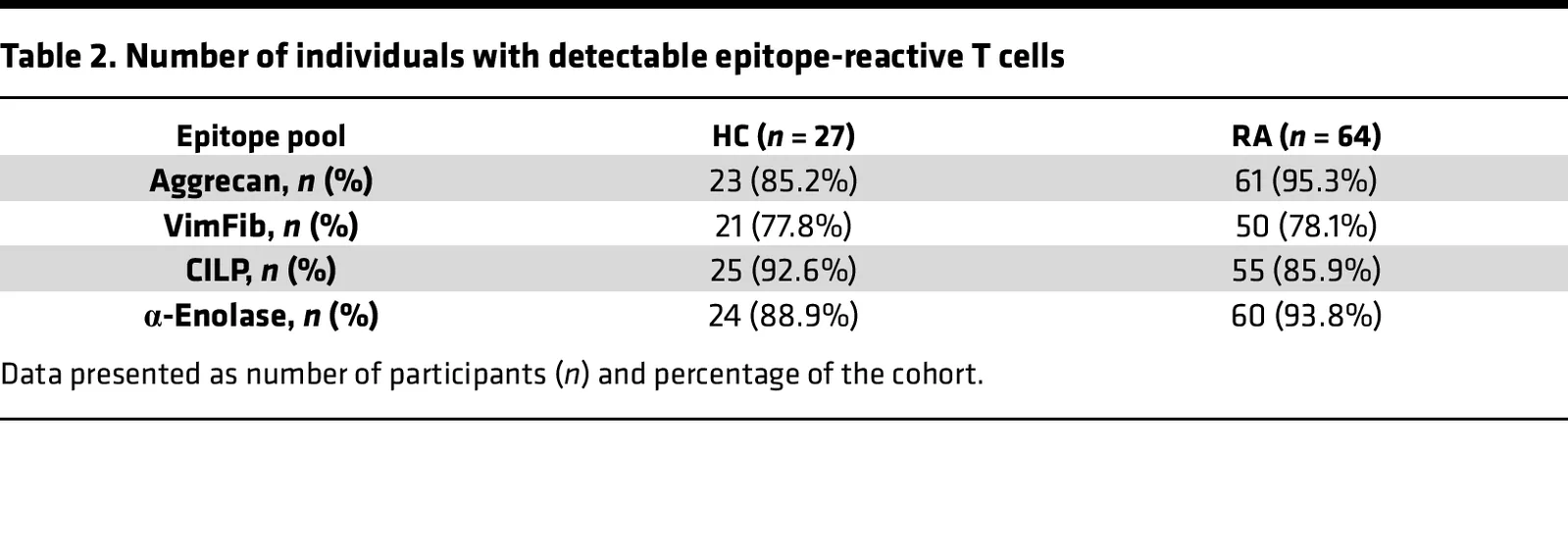
Number of individuals with detectable epitope-reactive T cells

**Table 3:**
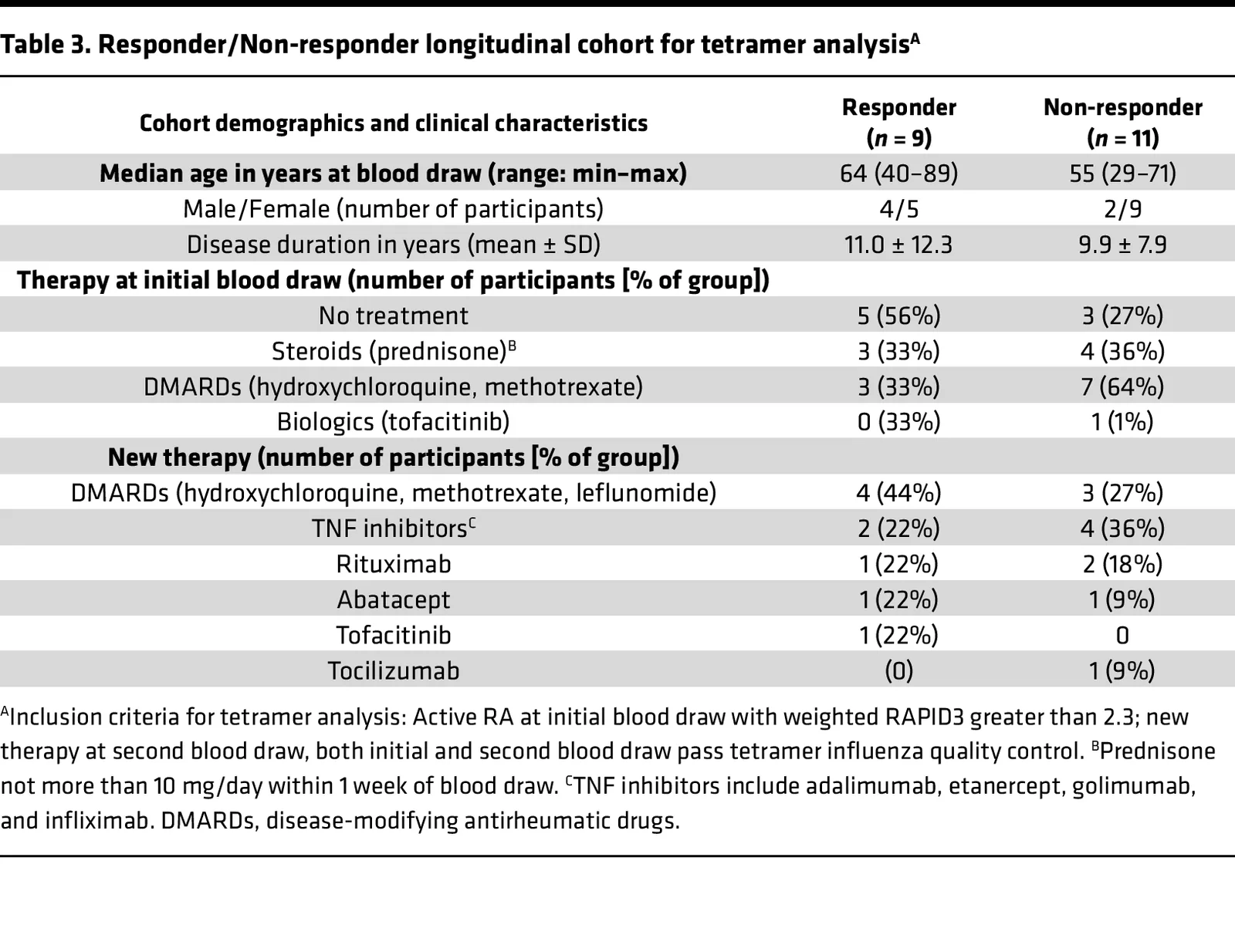
Responder/Non-responder longitudinal cohort for tetramer analysis^A^

## Version 1 09/08/2026

Electronic publication

## References

[1] https://iris.who.int/server/api/core/bitstreams/35a591a8-9bf5-4bbe-a7ff-8612ea36fa7f/content.

[2] Nguyen H, James EA (2016). Immune recognition of citrullinated epitopes. Immunology.

[3] Malmstrom V (2017). The immunopathogenesis of seropositive rheumatoid arthritis: from triggering to targeting. Nat Rev Immunol.

[4] Deane KD, Holers VM (2019). The natural history of rheumatoid arthritis. Clin Ther.

[5] Hill JA (2008). Arthritis induced by posttranslationally modified (citrullinated) fibrinogen in DR4-IE transgenic mice. J Exp Med.

[6] Thiele GM (2012). Citrullinated mouse collagen administered to DBA/1J mice in the absence of adjuvant initiates arthritis. Int Immunopharmacol.

[7] Gregersen PK (1987). The shared epitope hypothesis. An approach to understanding the molecular genetics of susceptibility to rheumatoid arthritis. Arthritis Rheum.

[8] Raza K (2005). Early rheumatoid arthritis is characterized by a distinct and transient synovial fluid cytokine profile of T cell and stromal cell origin. Arthritis Res Ther.

[9] Firestein GS (2003). Evolving concepts of rheumatoid arthritis. Nature.

[10] Kremer JM (2003). Treatment of rheumatoid arthritis by selective inhibition of T-cell activation with fusion protein CTLA4Ig. N Engl J Med.

[11] Elliott SE (2018). Affinity maturation drives epitope spreading and generation of proinflammatory anti-citrullinated protein antibodies in rheumatoid arthritis. Arthritis Rheumatol.

[12] Kaibara N (2008). Comparative histopathological analysis between tenosynovitis and joint synovitis in rheumatoid arthritis. Histopathology.

[13] Jonsson AH (2022). Granzyme K^+^ CD8 T cells form a core population in inflamed human tissue. Sci Transl Med.

[14] Bhattacharya D (2024). Single-cell characterisation of tissue homing CD4^+^ and CD8^+^ T cell clones in immune-mediated refractory arthritis. Mol Med.

[15] Dunlap G (2024). Clonal associations between lymphocyte subsets and functional states in rheumatoid arthritis synovium. Nat Commun.

[16] James EA (2014). Citrulline-specific Th1 cells are increased in rheumatoid arthritis and their frequency is influenced by disease duration and therapy. Arthritis Rheumatol.

[17] Pieper J (2018). Memory T cells specific to citrullinated α-enolase are enriched in the rheumatic joint. J Autoimmun.

[18] Song J (2021). Shared recognition of citrullinated tenascin-C peptides by T and B cells in rheumatoid arthritis. JCI Insight.

[19] Yamada H (2011). Preferential accumulation of activated Th1 cells not only in rheumatoid arthritis but also in osteoarthritis joints. J Rheumatol.

[20] Jang S (2022). Rheumatoid arthritis: pathogenic roles of diverse immune cells. Int J Mol Sci.

[21] Inamo J (2025). Deep immunophenotyping reveals circulating activated lymphocytes in individuals at risk for rheumatoid arthritis. J Clin Invest.

[22] Gerstner C (2020). Multi-HLA class II tetramer analyses of citrulline-reactive T cells and early treatment response in rheumatoid arthritis. BMC Immunol.

[23] Uchtenhagen H (2016). Efficient ex vivo analysis of CD4^+^ T-cell responses using combinatorial HLA class II tetramer staining. Nat Commun.

[24] Rims C (2019). Citrullinated aggrecan epitopes as targets of autoreactive CD4^+^ T cells in patients with rheumatoid arthritis. Arthritis Rheumatol.

[25] Rims C (2025). Antigen-specific T-cell frequency and phenotype mirrors disease activity in DRB1*04:04^+^ rheumatoid arthritis patients. Clin Exp Immunol.

[26] Kwok WW (2012). Frequency of epitope-specific naive CD4(+) T cells correlates with immunodominance in the human memory repertoire. J Immunol.

[27] Pincus T (2008). An index of only patient-reported outcome measures, routine assessment of patient index data 3 (RAPID3), in two abatacept clinical trials: similar results to disease activity score (DAS28) and other RAPID indices that include physician-reported measures. Rheumatology (Oxford).

[28] Pincus T (2008). RAPID3 (Routine Assessment of Patient Index Data 3), a rheumatoid arthritis index without formal joint counts for routine care: proposed severity categories compared to disease activity score and clinical disease activity index categories. J Rheumatol.

[29] Wiedeman AE (2020). Autoreactive CD8^+^ T cell exhaustion distinguishes subjects with slow type 1 diabetes progression. J Clin Invest.

[30] Levine JH (2015). Data-driven phenotypic dissection of AML reveals progenitor-like cells that correlate with prognosis. Cell.

[31] Sokolove J (2012). Autoantibody epitope spreading in the pre-clinical phase predicts progression to rheumatoid arthritis. PLoS One.

[32] Hecht C (2015). Additive effect of anti-citrullinated protein antibodies and rheumatoid factor on bone erosions in patients with RA. Ann Rheum Dis.

[33] Jilani AA, Mackworth-Young CG (2015). The role of citrullinated protein antibodies in predicting erosive disease in rheumatoid arthritis: a systematic literature review and meta-analysis. Int J Rheumatol.

[34] Orr C (2017). Synovial immunophenotype and anti-citrullinated peptide antibodies in rheumatoid arthritis patients: relationship to treatment response and radiologic prognosis. Arthritis Rheumatol.

[35] Lundberg K (2005). Citrullinated proteins have increased immunogenicity and arthritogenicity and their presence in arthritic joints correlates with disease severity. Arthritis Res Ther.

[36] Sokolove J (2011). Immune complexes containing citrullinated fibrinogen costimulate macrophages via Toll-like receptor 4 and Fcγ receptor. Arthritis Rheum.

[37] Kinloch A (2005). Identification of citrullinated alpha-enolase as a candidate autoantigen in rheumatoid arthritis. Arthritis Res Ther.

[38] Kim HY (1999). Enhanced T cell proliferative response to type II collagen and synthetic peptide CII (255-274) in patients with rheumatoid arthritis. Arthritis Rheum.

[39] Law SC (2012). T-cell autoreactivity to citrullinated autoantigenic peptides in rheumatoid arthritis patients carrying HLA-DRB1 shared epitope alleles. Arthritis Res Ther.

[40] von Delwig A (2010). Response of Th17 cells to a citrullinated arthritogenic aggrecan peptide in patients with rheumatoid arthritis. Arthritis Rheum.

[41] Alexei vD, James L H (2010). Response of Th17 cells to a citrullinated arthritogenic aggrecan peptide in patients with rheumatoid arthritis. Arthritis Rheum.

[42] James EA (2010). HLA-DR1001 presents “altered-self” peptides derived from joint-associated proteins by accepting citrulline in three of its binding pockets. Arthritis Rheum.

[43] Carmona-Rivera C (2017). Synovial fibroblast-neutrophil interactions promote pathogenic adaptive immunity in rheumatoid arthritis. Sci Immunol.

[44] Gaffen SL (2009). The role of interleukin-17 in the pathogenesis of rheumatoid arthritis. Curr Rheumatol Rep.

[45] Fonseka CY (2018). Mixed-effects association of single cells identifies an expanded effector CD4^+^ T cell subset in rheumatoid arthritis. Sci Transl Med.

[46] Pawlik A (2003). The expansion of CD4^+^CD28^–^ T cells in patients with rheumatoid arthritis. Arthritis Res Ther.

[47] Rao DA (2017). Pathologically expanded peripheral T helper cell subset drives B cells in rheumatoid arthritis. Nature.

[48] Moon JS (2023). Cytotoxic CD8^+^ T cells target citrullinated antigens in rheumatoid arthritis. Nat Commun.

[49] Patel DD (2001). CXCR3 and CCR5 ligands in rheumatoid arthritis synovium. Clin Immunol.

[50] Sohrabian A (2018). Number of individual ACPA reactivities in synovial fluid immune complexes, but not serum anti-CCP2 levels, associate with inflammation and joint destruction in rheumatoid arthritis. Ann Rheum Dis.

[51] Li Y (2024). Stem-like T cells are associated with the pathogenesis of ulcerative colitis in humans. Nat Immunol.

[52] Sato Y (2023). Stem-like CD4^+^ T cells in perivascular tertiary lymphoid structures sustain autoimmune vasculitis. Sci Transl Med.

[53] James EA (2023). Multifaceted immune dysregulation characterizes individuals at-risk for rheumatoid arthritis. Nat Commun.

[54] Novak EJ (1999). MHC class II tetramers identify peptide-specific human CD4(+) T cells proliferating in response to influenza A antigen. J Clin Invest.

[55] Hahne F (2009). flowCore: a Bioconductor package for high throughput flow cytometry. BMC Bioinformatics.

[56] van der Maaten LJP, Hinton GE (2008). Visualizing high-dimensional data using t-SNE. J Mach Learn Res.

[57] Maaten LJPvd (2014). Accelerating t-SNE using Tree-based algorithms. J Mach Learn Res.

[58] https://github.com/jkrijthe/Rtsne.

[59] Chen H (2016). Cytofkit: a Bioconductor package for an integrated mass cytometry data analysis pipeline. PLoS Comput Biol.

[60] Becher B (2014). High-dimensional analysis of the murine myeloid cell system. Nat Immunol.

[61] Wong MT (2015). Mapping the diversity of follicular helper T cells in human blood and tonsils using high-dimensional mass cytometry analysis. Cell Rep.

[62] Gu Z (2016). Complex heatmaps reveal patterns and correlations in multidimensional genomic data. Bioinformatics.

