# Supplementary material for: Specificity, frequency, and phenotype of citrullinated-specific T cells vary with disease activity in rheumatoid arthritis
Source: JCI Insight. 2026 Sep 8;11(17):e202159. doi: 10.1172/jci.insight.202159 (PMC13564074; doi:10.1172/jci.insight.202159)
Supplement: Supplementary file 1 [file jciinsight-11-202159-s024.pdf]

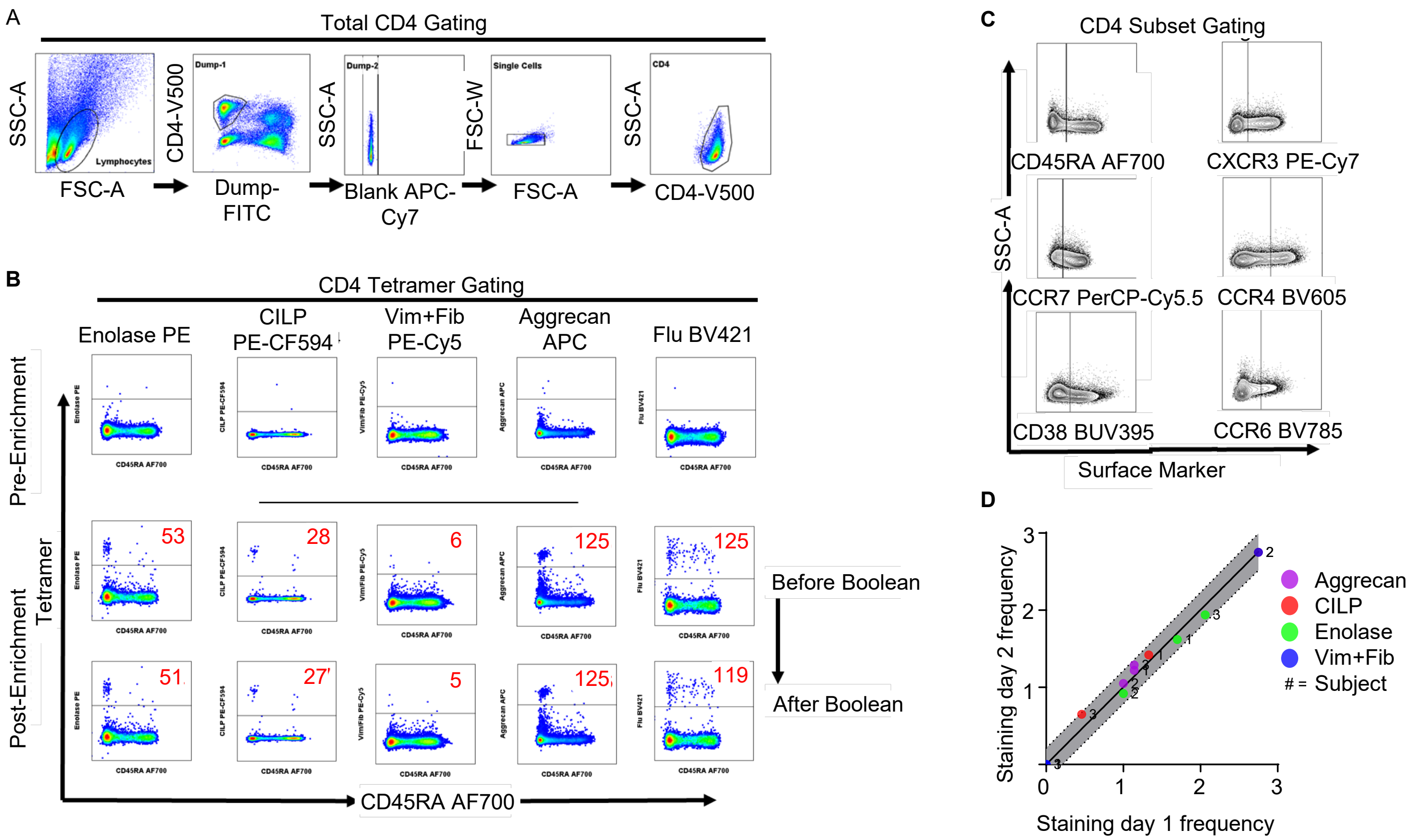

**Boolean Gating Selection**	
Tetramer	Colors included and excluded
Enolase	PE+ & CF594- & Cy5- & APC- & BV421-
CILP	CF594+ & PE- & Cy5- & APC- & BV421-
Aggrecan	APC+ & PE- & CF594- & Cy5- & BV421-
Vim & Fib	Cy5+ & PE- & CF594- & APC- & BV421-
Influenza	BV421+ & PE- & CF594- & Cy5- & APC-

Supplemental Figure 1. Tetramer assay. (A) Representative FACS plots depicting gating scheme for total CD4$^+$ T cell selection**. (B)** Representative FACS plots depicting CD4$^+$ parent population analyzed for CD4 tetramer cells using pre-enrichment sample along with Boolean gating of post-enrichment sample to select single-color tetramer cells. Red numbers indicate the raw tetramer count before and after Boolean gating. **(C)** CD4$^+$ parent population used to gate phenotypic surface markers. (**D)** Reproducibility of individual antigen tetramer staining in 3 different patients indicated by colors and numbers respectively. The same sample was stained on two different days (X and Y axis are tetramer frequency per million CD4$^+$ T cells each day). Shading within bands are 95% confidence of the best fit line using simple linear regression.

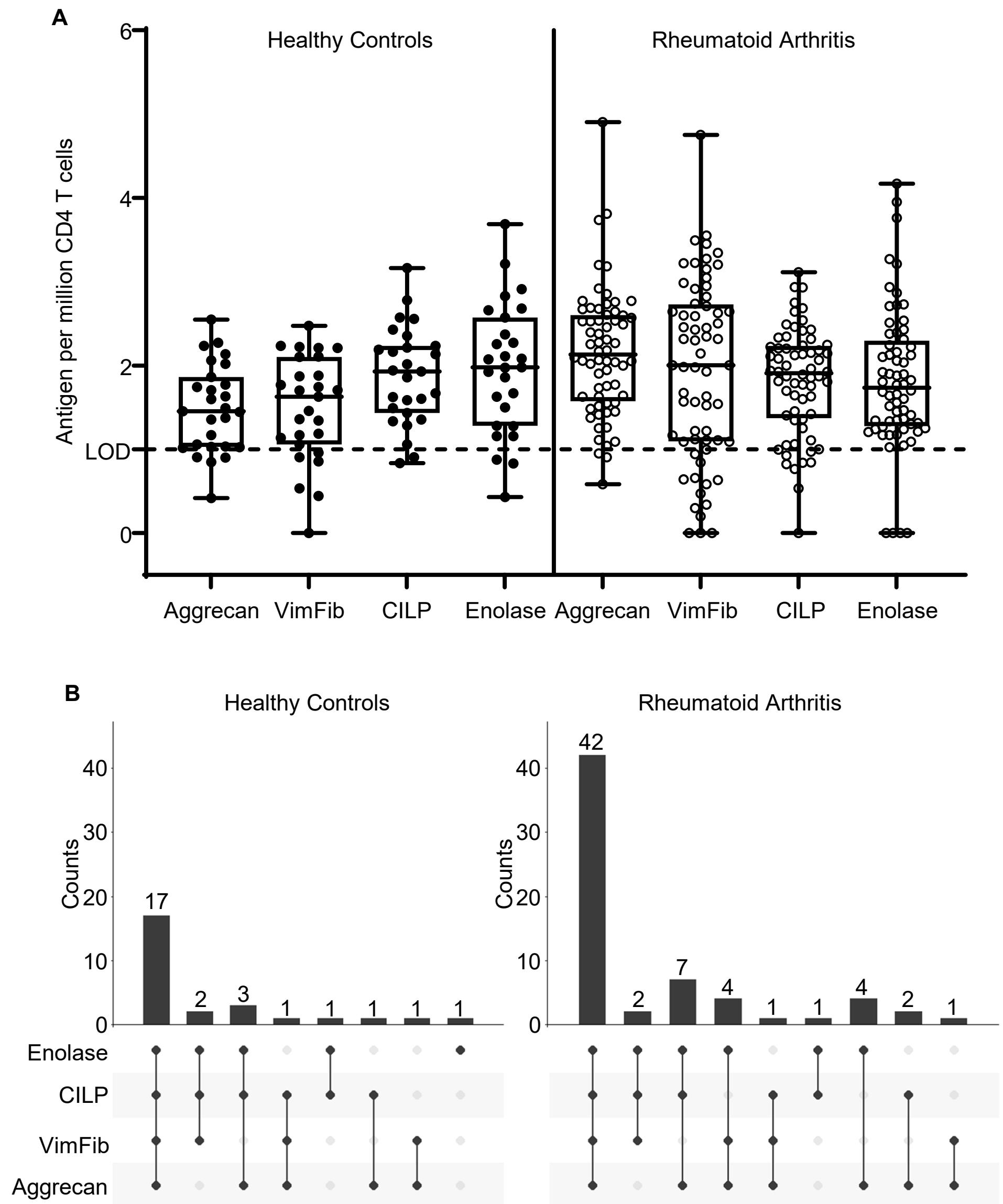

Supplemental Figure 2. The frequency of cit-aggrecan, VimFib, CILP, and enolase-specific CD4+ T cells were evaluated for presence and multiplicity among RA and healthy subjects. (A) Observed frequencies of tetramer labeled cells for each antigen pool referenced against the limit of detection (LOD, 1.0 cell per million CD4+ T cells) for healthy subjects (filled circles, n=27) and individuals with RA (open circles, n=64), frequency was log2(x+1) transformed **(B)** Upset plots that depict subject counts based on number of antigens detected.

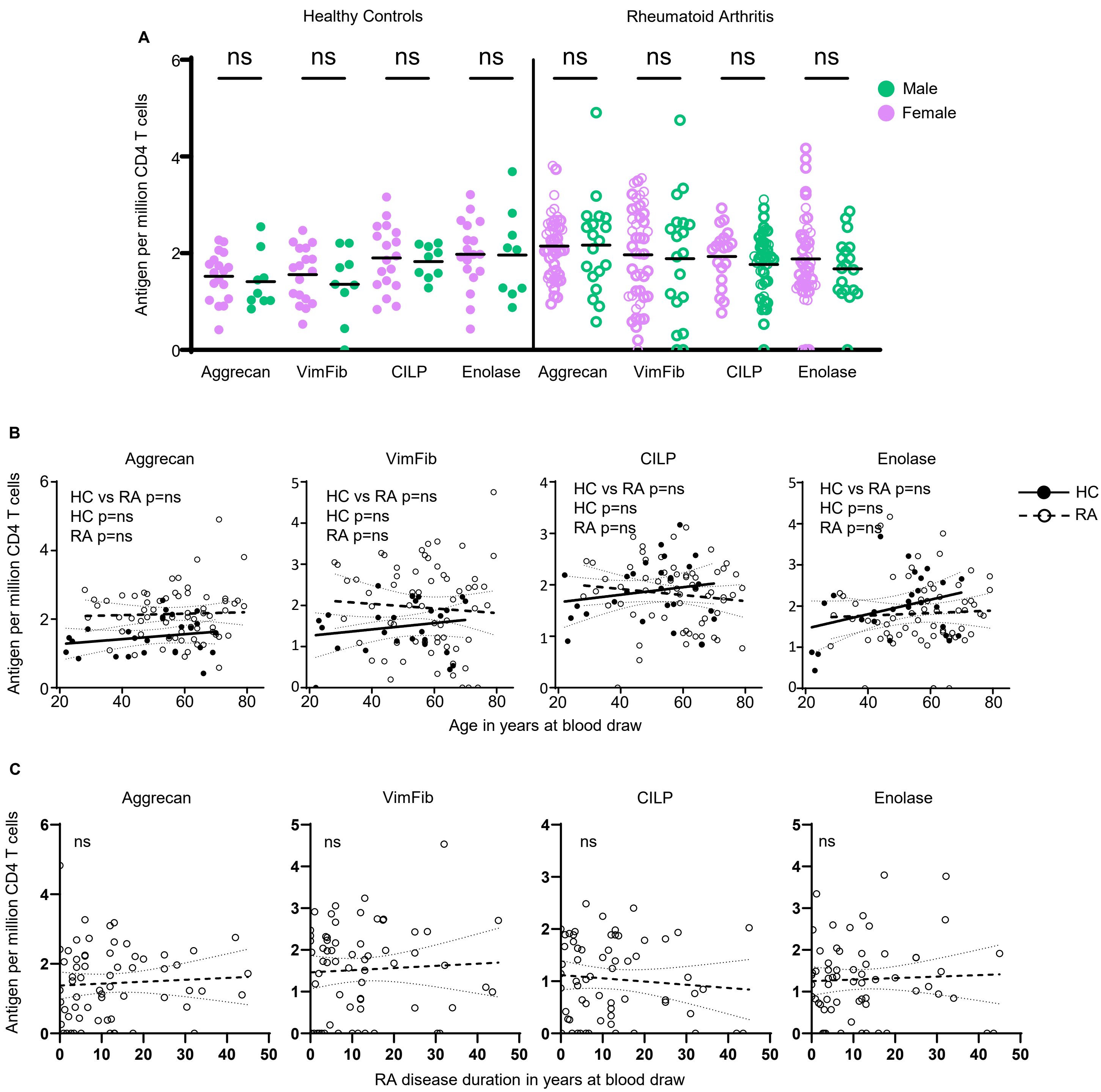

Supplemental Figure 3. The influence of biological sex, age, and RA disease duration on the frequency of cit-aggrecan, VimFib, CILP, and enolase-specific CD4$^+$ T cells in healthy and rheumatoid arthritis participants. (A) Cit-specific CD4$^+$ T cell frequencies based on biological sex for rheumatoid arthritis (RA; n=64) and healthy control (HC; n=27) cohorts; line indicates median, frequency was log2(x+1) transformed and 2-way nonparametric Wilcoxon was used to compare males and females where NS indicates a p-value greater than 0.05. **(B)** Correlation between cit-specific T cell frequencies for RA (n=64) or HC (n=27) subjects and age: frequency was log2(x+1) transformed, nonparametric Spearman test was used to correlate frequency and age, and simple linear regression was used to compare slopes of healthy versus RA respectively, for each graph NS indicates a p-value greater than 0.05. **(C)** Correlation between cit T cell frequencies for RA and disease duration at draw are shown for individual RA subjects (N=64) where nonparametric Spearman test was used and NS indicates a p-value greater than 0.05.

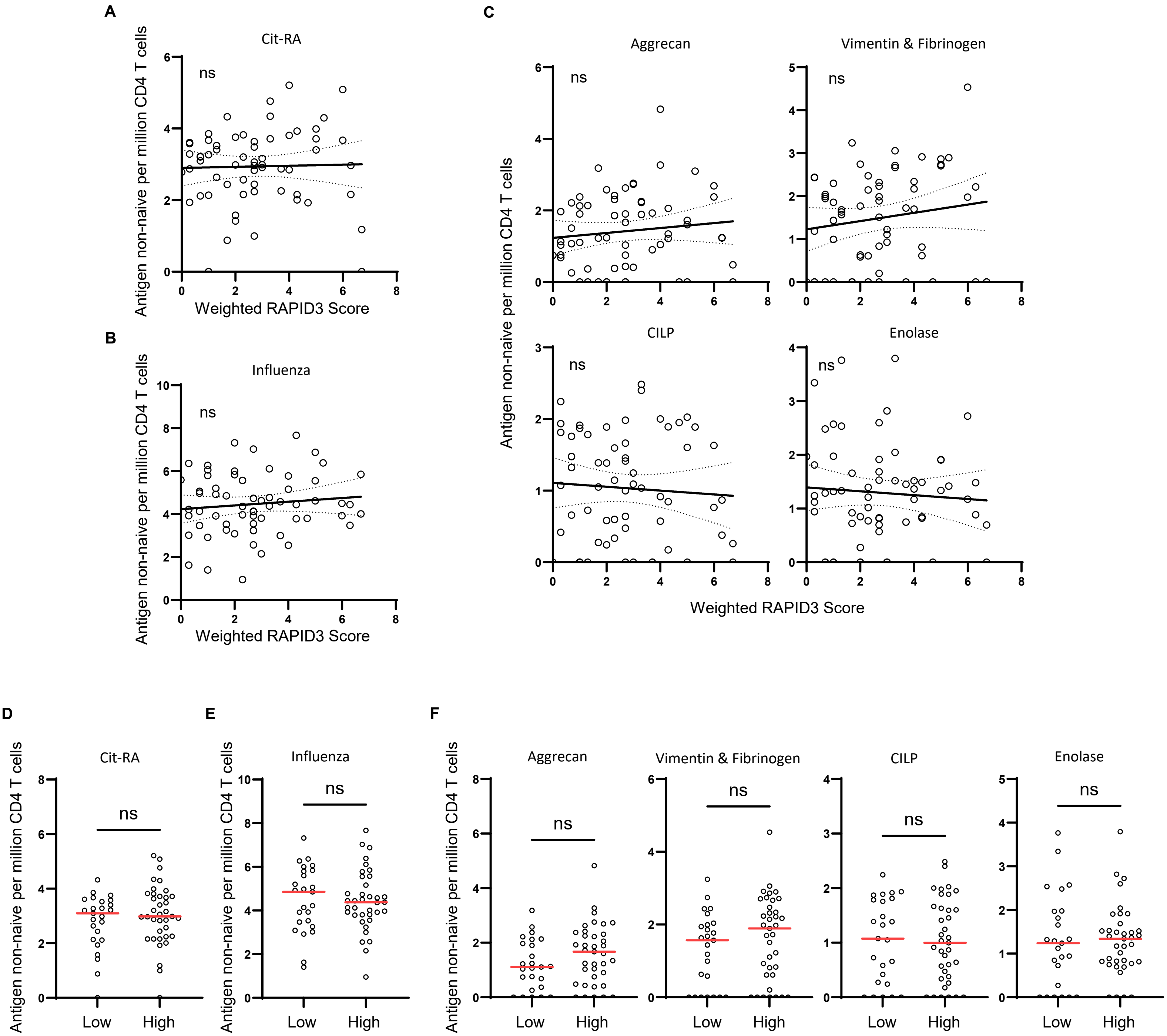

Supplemental Figure 4. The cit-reactive frequency does not correlate by weighted RAPID3 response Antigen frequency was tested for correlation against weighted RAPID3 by either continuous (A-C) or categorical groups (those RA subjects' wRAPID3 <2.3 being "low" and wRAPID3 >2.3 being "high"; D-F). **(A-C)** The non-naïve antigen frequency (y-axis) are plotted for each RA subject (N=62) against their wRAPID3 (x-axis) where **c**orrelation was determined using nonparametric spearman method where the two-tailed p-value is shown within each respective graph (NS indicate a p-value greater than 0.05). **(D-F)** The non-naïve antigen frequency of RA subjects grouped by either wRAPID3 activity low (N=25) or high (N=37) are compared by nonparametric Kruskal-Wallis with Dunn's multiple comparisons where the p-value is displayed for each respective graph (NS indicate a p-value greater than 0.05, red lines are median). Non-naïve antigen frequency was Log2(x +1) transformed for all graphs (A-F).

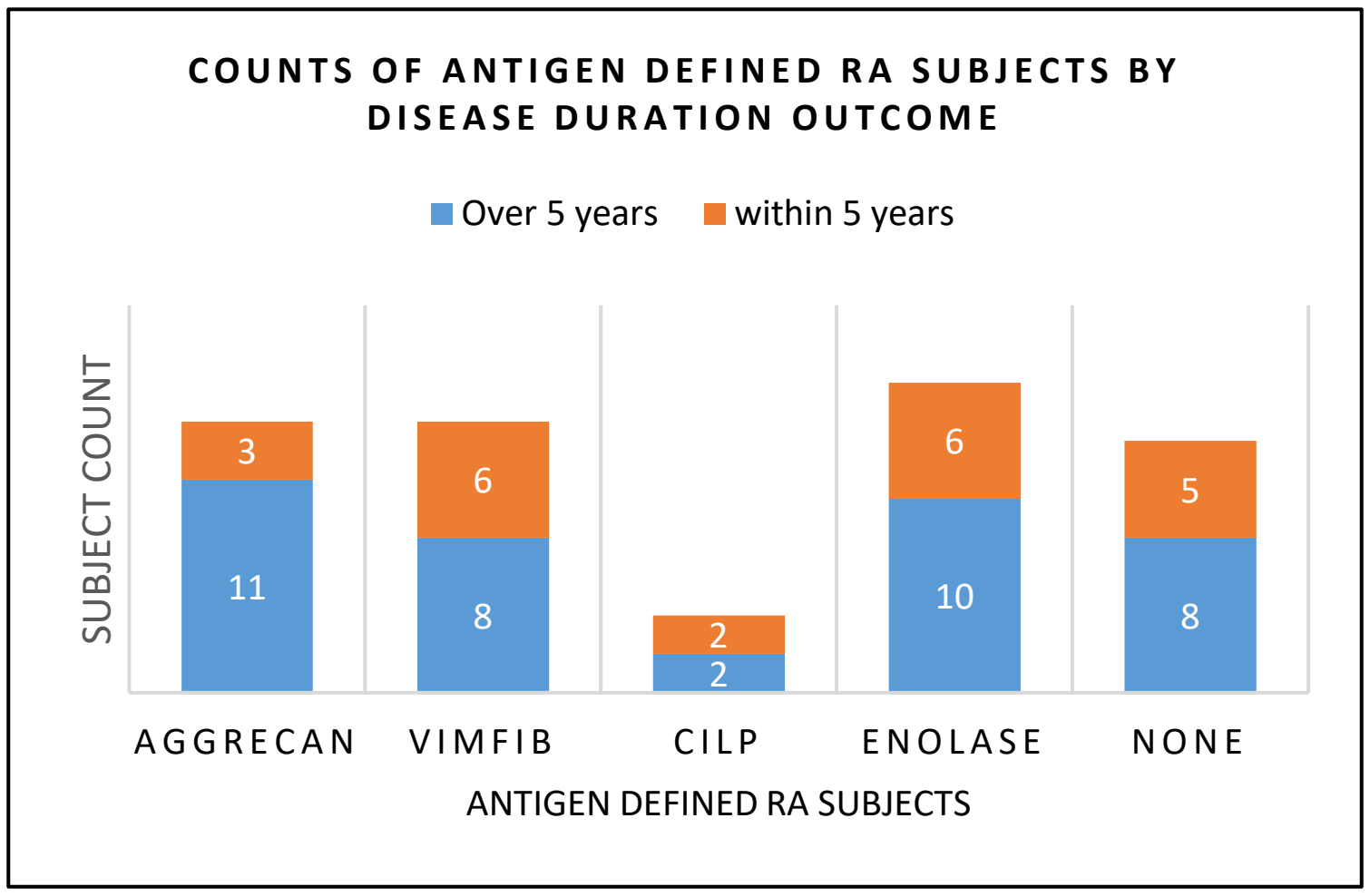

Supplemental Figure 5. Cit-antigens do not define RA subjects based on disease duration. Bar graph depicting antigen defined RA subjects that were grouped based on RA disease duration of within 5 years or over 5 years.

A

Descriptive frequencies of antigen defined RA subjects and disease activity							
Antigen that defines a subject	**Aggrecan**	**VimFib**	**CILP**	**Enolase**	**not-defined**	Total	
**Inactive RA** Near-Remission + Low wRAPID3	**3**	**2**	**1**	**8**	**10**	**24**	Count
	5.1	3.4	1.7	13.6	17.0	40.7	Total %
	23.1	14.3	25.0	50.0	83.3		Column %
	12.5	8.3	4.2	33.3	41.7		Row %
**Active RA** High + Moderate wRAPID3	**10**	**12**	**3**	**8**	**2**	**35**	Count
	17.0	20.3	5.1	13.6	3.4	59.3	Total %
	76.9	85.7	75.0	50.0	16.7		Column %
	28.6	34.3	8.6	22.9	5.7		Row %
Total	13	14	4	16	12	59	
	22.0	23.7	6.8	27.1	20.3		

B

**Fisher's exact test p-values (citAg defined vs undefined RA subjects)**	
Aggrecan	0.0048
VimFib	0.0011
CILP	0.0632
Enolase	0.1144

Supplemental Figure 6. RA subjects with active RA are defined by cit-aggrecan or vimentin & fibrinogen. (A) Table outlining frequencies based on different groups. **(C)** Table of Fisher's exact test p-values where those subjects defined by an antigen were compared to those not-antigen-defined**.** In total, 24 RA subjects were considered "inactive RA activity" and 35 "active RA" at the time of draw (total N = 59). "Inactive RA" are those RA subjects who's weighted RAPID3 is less than 2.3 and "Active RA" are those who's weighted RAPID3 is greater than 2.3 respectively.

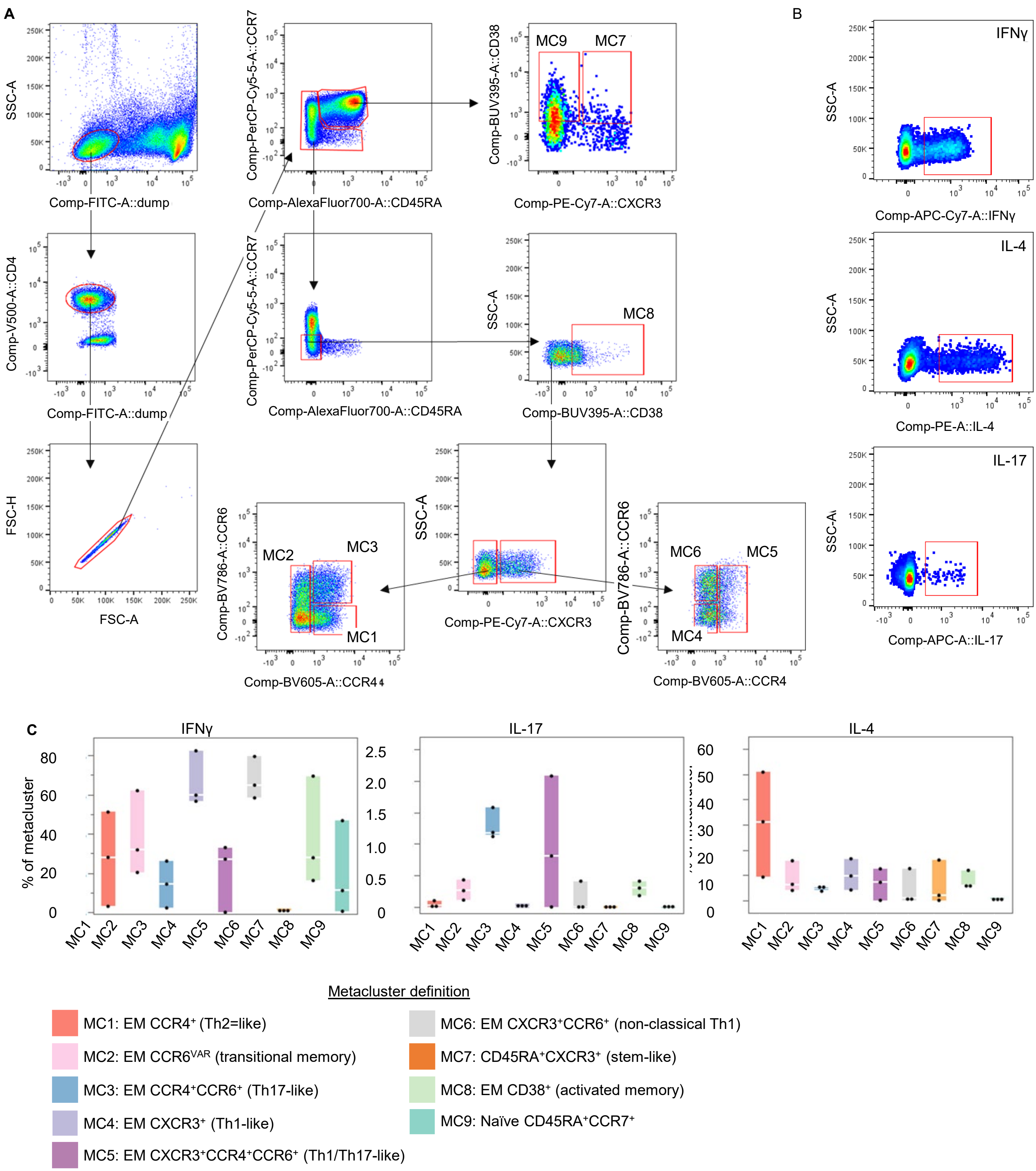

Supplemental Figure 7. Manual gating to select metaclusters and cytokine frequency. (A) Representative FACS plots depicting gating scheme for metaclusters 1-9. **(B)** Representative gating to calculate the frequency of interferon-gamma, IL-4, and IL-17 expression for each manually selected metacluster. **(C)** The frequency of interferon-gamma, IL-4, and IL-17 expression analyzed for T cells falling within each metacluster from three RA subjects.

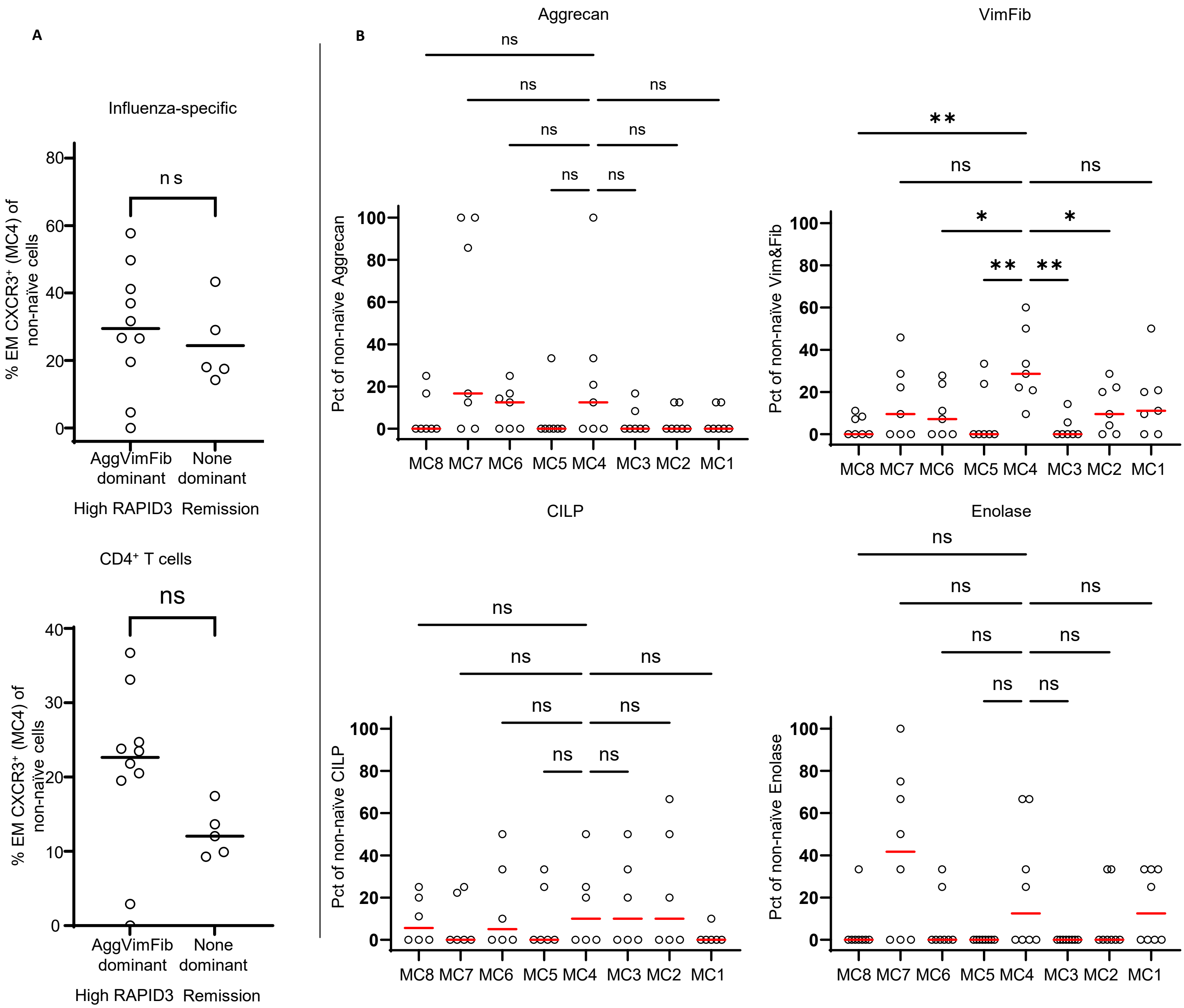

Supplemental Figure 8. Among diverse cit-specific T cell phenotypes, distinct clusters are increased in active RA. (**A**) EM CXCR3 percent of non-naïve cellsubset are shown for each individual by influenza and CD4T respectively among those subjects defined by aggrecan,vimentin&fibrinogen with high wRAPID3 disease activity (N=10) or those not-defined in wRAPID3 remission (N=5), statistics shown is 2-way nonparametric Wilcoxon test. (**B**) The predominant cit-antigen phenotype of those high RAPID3 RA subjects are evaluated using a linear mixed model. The phenotype frequency of each metacluster is plotted for individual cit-antigen respectively for those RA subjects with high disease activity as defined by weighted RAPID3 (wRAPID3 > 4.0). A mixed effect analysis of multiple comparisons was used to calculate statistical comparisons shown within the graphs. The samples reported pass quality control as outlined in the Methods. Sample number aggrecan N=7, VimFib N=7, CILP N=6, and enolase N=8. Lines for both A and B are median.

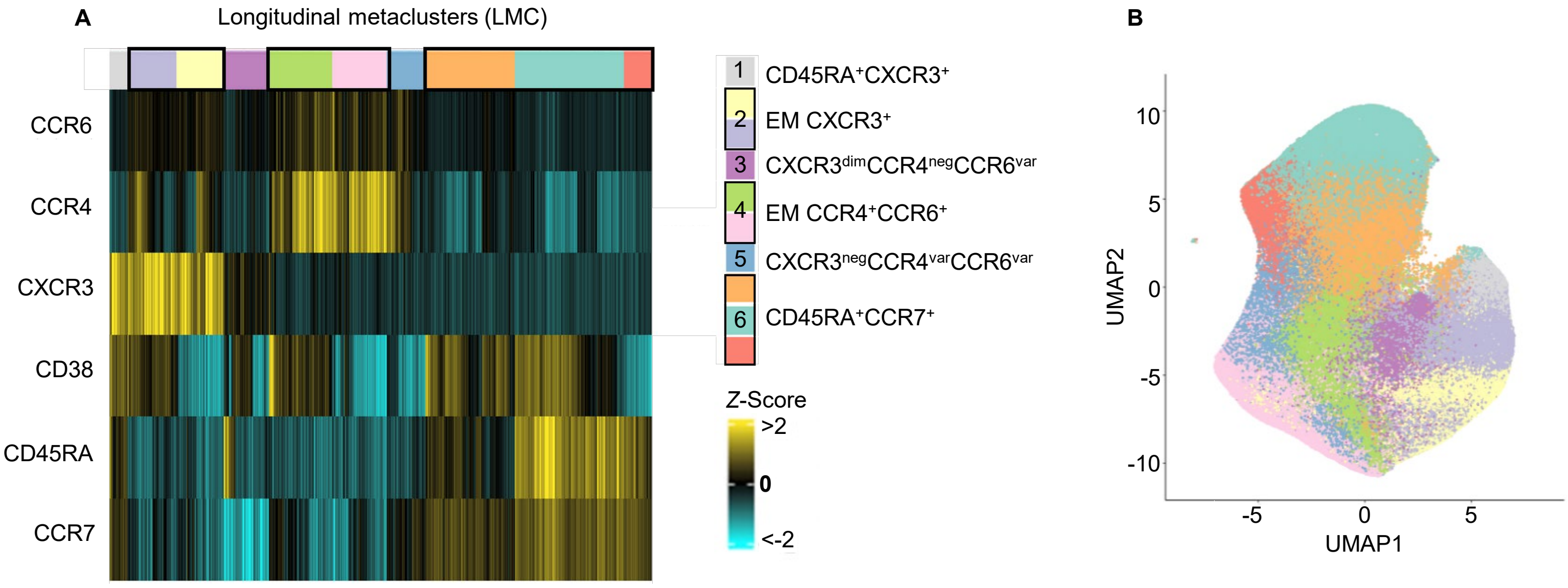

Supplemental Figure 9. CD4 T cell subsets determined by DISCOV-R. (A) Heat map showing all CD4^{+} T cell phenotypes from 108 samples originating from 37 RA subjects, hierarchically clustered by expression of six phenotyping markers as a z-score comparing mean cluster intensity to total CD4^{+} T cell intensity for each subject. The resulting dendrogram is sliced into 10 metaclusters (MC) with color bar across top indicating MC assignment respectively. Note that boxes within the color bar are metaclusters that were combined based on distance and overlap within the UMAP. **(B)** UMAP depicting bulk CD4^{+} T cell landscape and the locations of each assigned cluster.

A

Number of subjects with detected aggrecan, vimentin & fibrinogen by cohort (LOD ≥ 1.0)		
Cohort	Cross Sectional (CS) N=64	Longitudinal Baseline (LB) N=30
Aggrecan	61 (95.3%)	28 (93.3%)
Vimentin & Fibrinogen	50 (78.1%)	25 (83.3%)

B

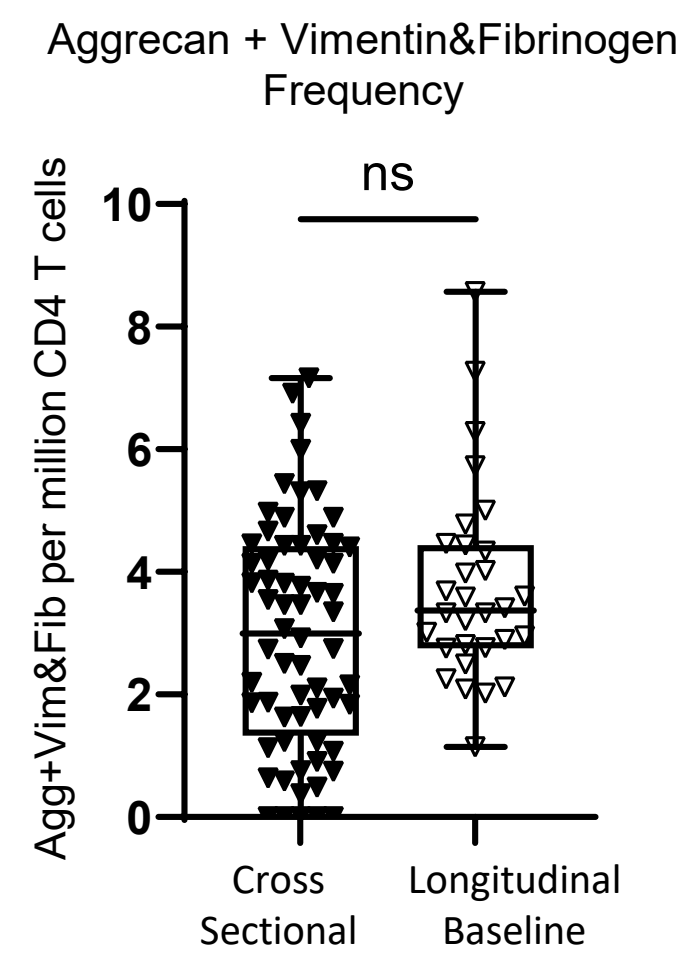

C

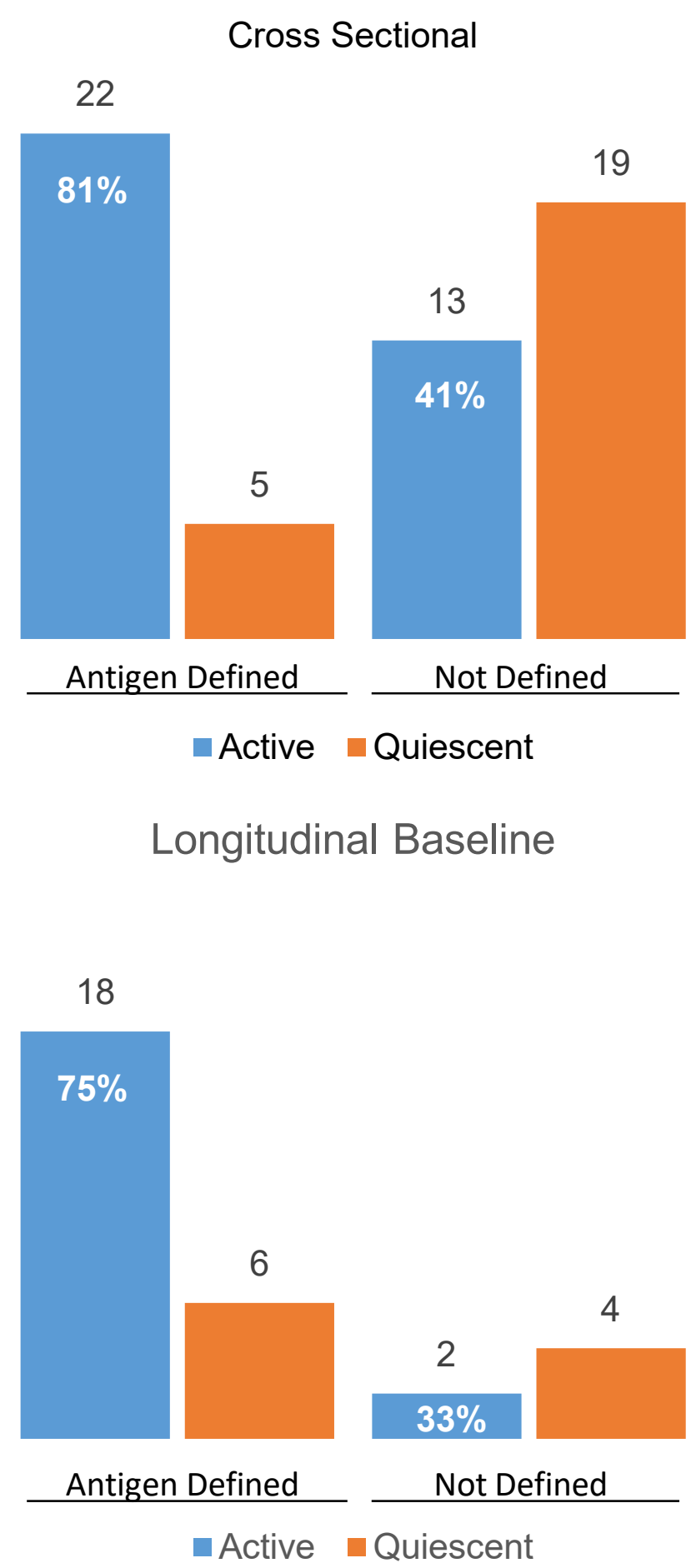

D

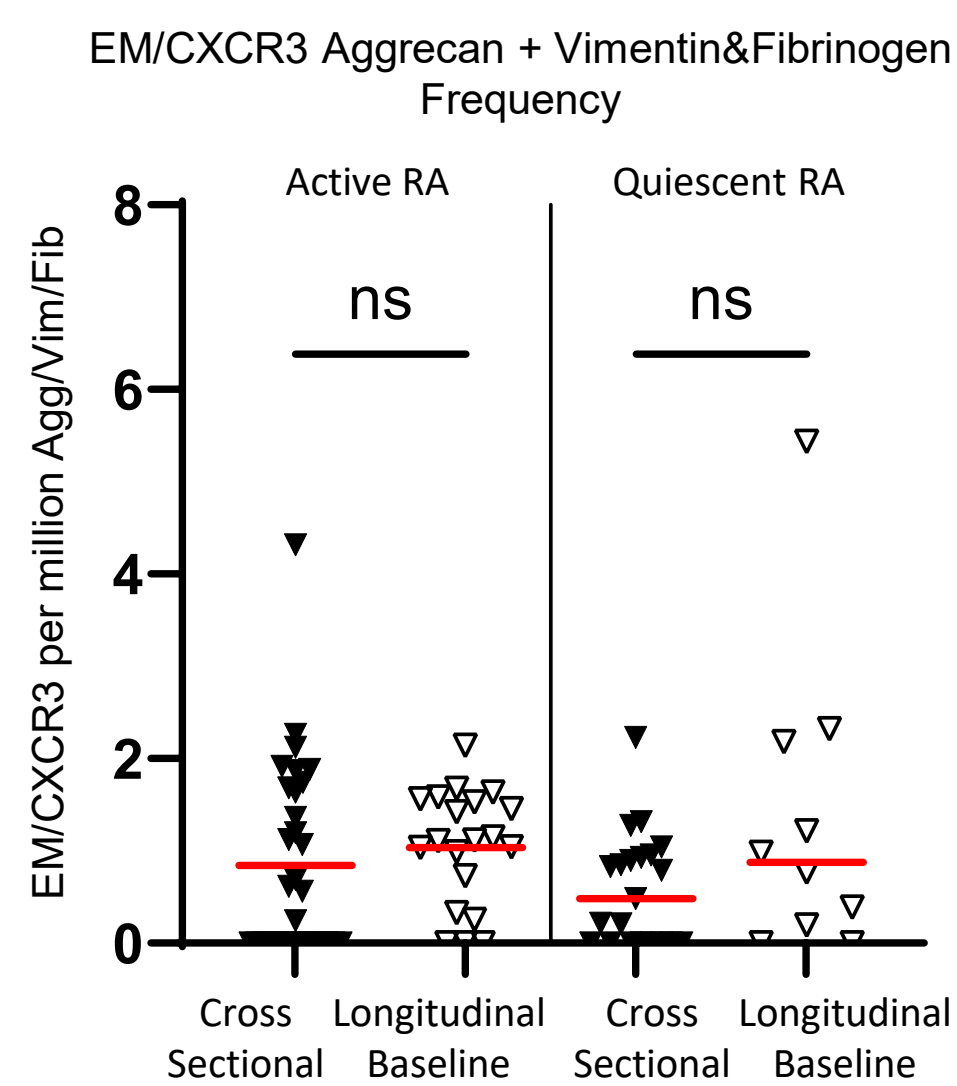

Supplemental Figure 10. Aggrecan, vimentin, and fibrinogen findings are shared between cross sectional and longitudinal cohorts. Citrulline antigens aggrecan, vimentin, and fibrinogen were used to compare the cross-sectional and longitudinal cohorts. **(A)** The number of samples where aggrecan and VimFib were detected among the cross-sectional (CS, N=64) and longitudinal baseline (LB, N=30) cohorts where detection was defined as the limit of detection (LOD) ≥ 1.0 cells per million CD4. **(B)** The frequency of concatenated aggrecan+VimFib for each subject is shown where frequency is reported as per million CD4 with y-axis transformed Log2(x+1). Two-tailed non-parametric Wilcoxon was used for statistical comparison. Whiskers are min to max, and line within boxes are the mean. **(C)** The number of RA subjects defined as either aggrecan+VimFib or those not-aggrecan+VimFib (method as outlined in figure 1D). The RA subjects were grouped as either having quiescent RA (orange colored, wRAPID3 ≤ 2.0) or active (blue colored, wRAPID3 ≥ 2.3) between the two cohorts. Percentages are number of subjects among antigen-defined or not-defined groups. (**D**) The frequency of EM CXCR3 aggrecan+VimFibmcells plotted for each subject by cohort and weighted RAPID3 (as outlined in panel C). Frequency is reported as per million CD4 where the y-axis is Log2(x+1) transformed. Samples reported are those whose parent event counts are at least 8; active-CS N=32, active-LB N=20, quiescent CS N=21, quiescent-LB N=10). Two tailed non-parametric Wilcoxon was used for statistical comparisons.

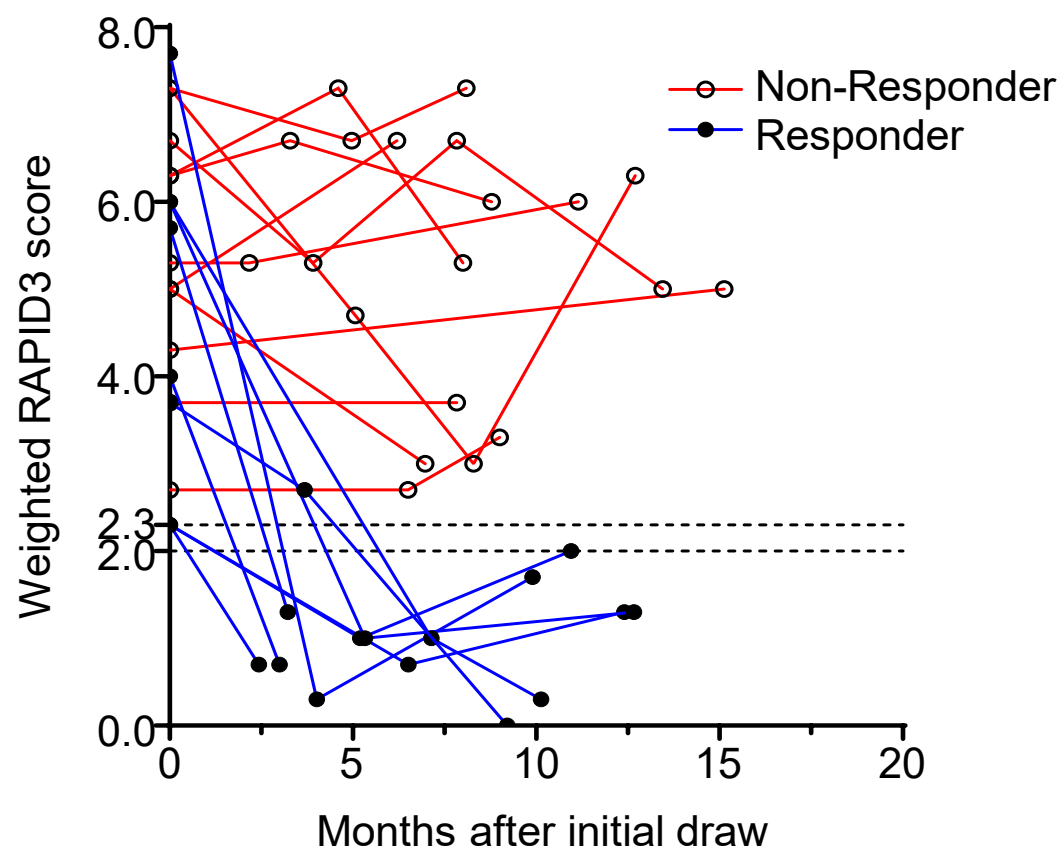

Supplemental Figure 11. Longitudinal Sampling of high and low disease activity subjects DR0401 positive individuals with seropositive RA who had high disease activity (defined by RAPID3 weighted score ≥ 2.3) and who were transitioning to a new therapy were sampled longitudinally after enrollment (months after draw shown on x-axis) and classified as responders (blue lines, filled data points) or non-responders (red lines, open data points) based on their final RAPID3 score (responder = decreased to < 2.3 after transition to new therapy; non-responder = maintained RAPID3 score ≥ 2.3).

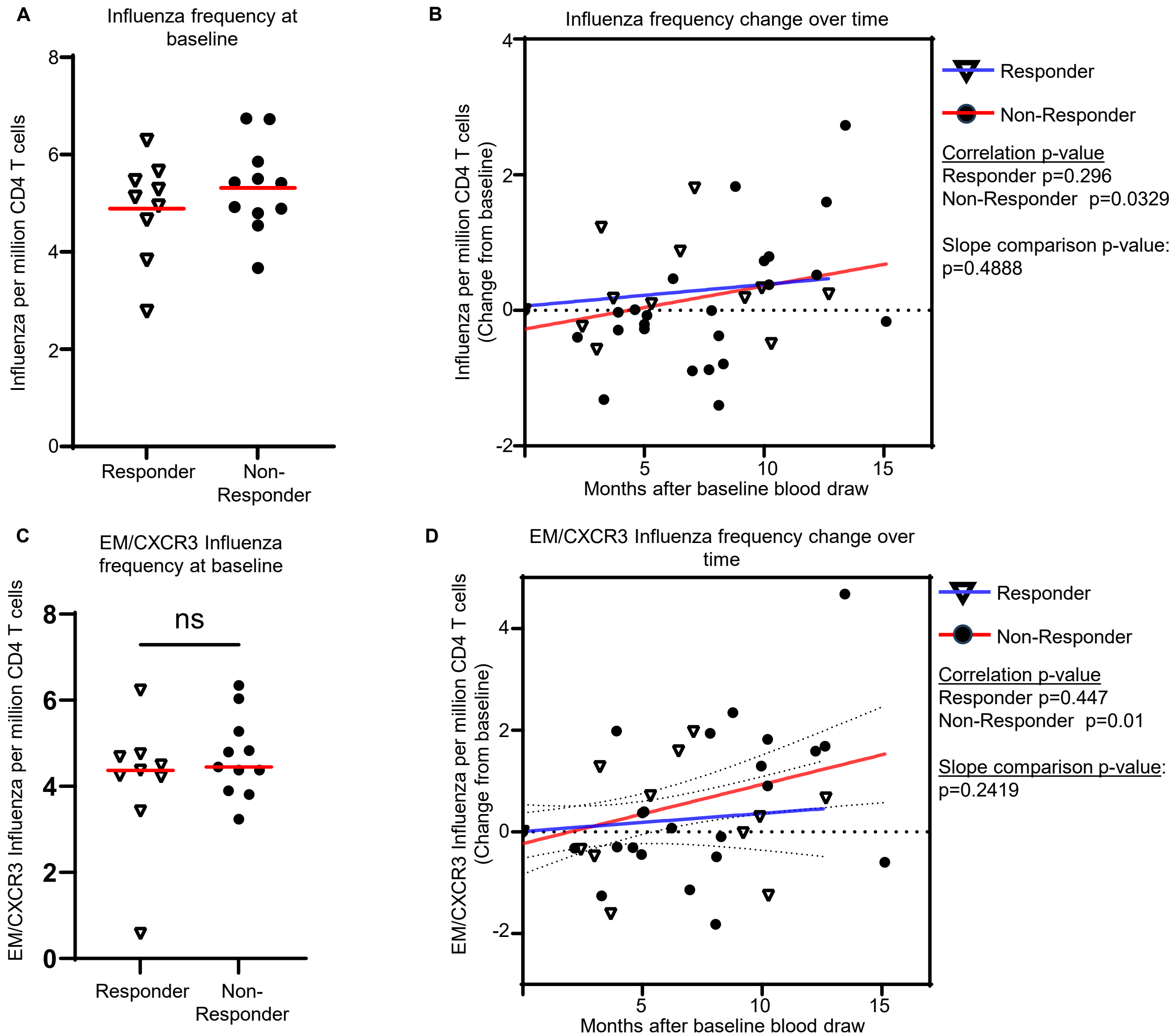

Supplemental Figure 12. Frequency of influenza-specific Th1-like cells RA individuals who respond to therapy. The frequency of influenza-specific from RA participants who had blood draws before and after starting a new therapy. At first draw, all individuals had active disease based on a weighted RAPID3 ≥2.3. These individuals were subsequently defined as Responder or Non-Responders based on their weighted RAPID3 at second draw (Responder RAPID3<2.0; Non-Responder RAPID3≥2.3) see **Supplemental Figure 11**. (**A**) Frequency of influenza-specific $CD4^+$ T cells in Responders in Responders (n=9) and Non-Responders (n=11); p-value calculated using two-tailed non-parametric Wilcoxon test (**B**) The frequency of influenza-specific $CD4^+$ T cells in Responders (n=20) and Non-Responders (n=33) was normalized to initial draw and then plotted against the number of months thereafter; simple linear regression model, 95% confidence intervals, and two-tailed Spearman's correlation. (**C**) The baseline frequency of cit-Th1-like (EM CXCR3+) influenza cells in responders (N=9) were compared to non-responders (N=11); p-value calculated using two-tailed non-parametric Wilcoxon test. (**D**) The frequency of cit-Th1-like (EM $CXCR3^+$) cells in Responders (n=20) and Non-Responders (n=33) was normalized to initial draw and then correlated with months after baseline draw prior to new therapy. non-parametric Spearman correlation was used to determine correlation to time and simple linear regression was used to determine slope differences between the two groups. For C and D datapoints are those samples where parent event count is at least 8 and shown in Figure 4. Graphs A-D are log2(x +1) transformed.

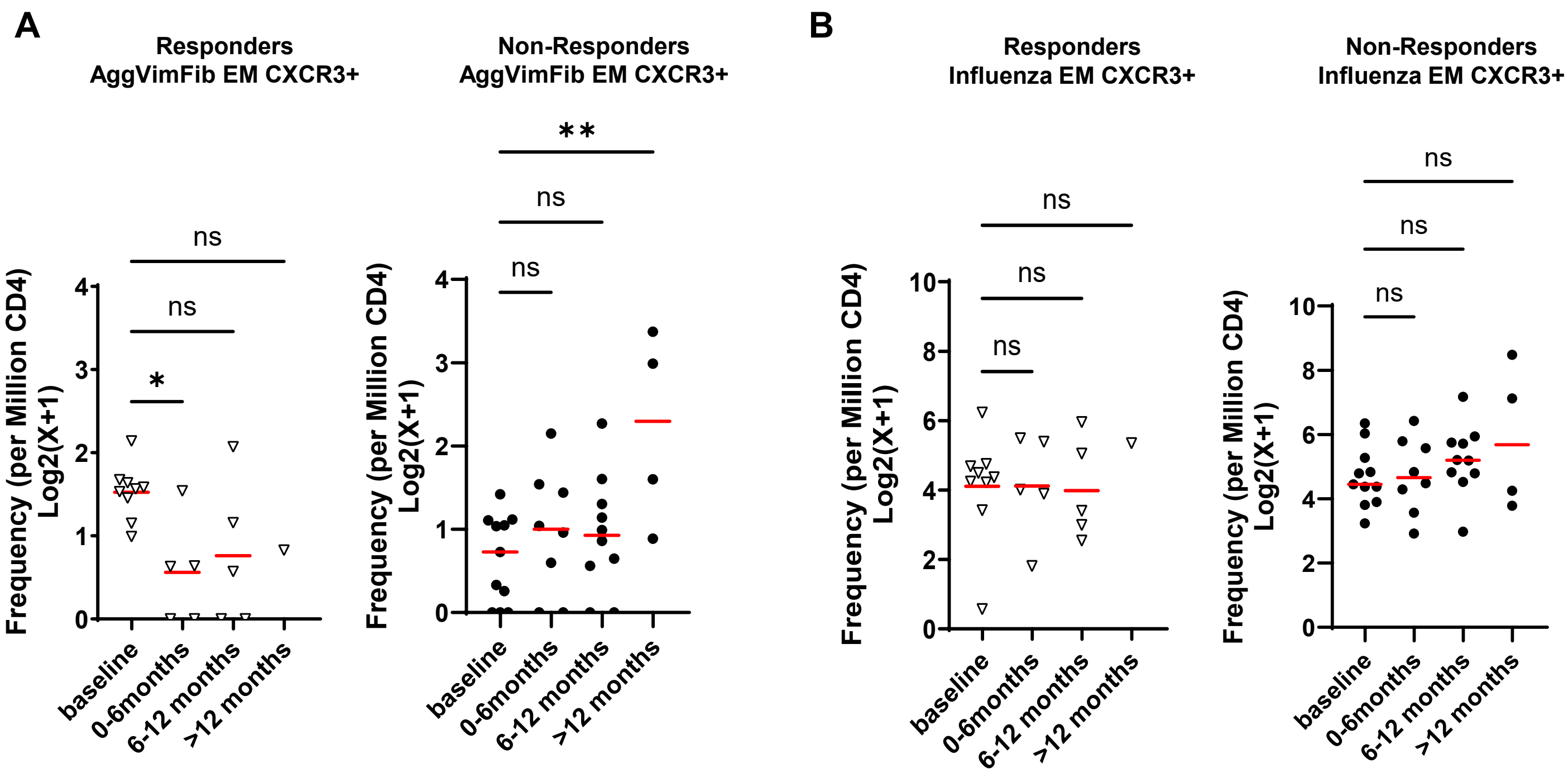

Supplemental Figure 13. The frequency of cit-specific Th1-like cells is decreased individuals who respond to therapy. The frequency of cit-aggrecan and cit-VimFib cells were combined from RA participants who had blood draws before and after starting a new therapy. At first draw, all individuals had active disease based on a weighted RAPID3 ≥2.3. These individuals were subsequently defined as Responder or Non-Responders based on their weighted RAPID3 at second draw (Responder RAPID3<2.0; Non-Responder RAPID3≥2.3) see **Supplemental Figure 11**. (**A**) The frequency of AggVimFib cit-Th1-like (EM $CXCR3^{+}$) cells in Responders before (Blood draw #1, n=9) and after therapy (Blood draw #2, n=5; Blood draw #3, n=5; Blood draw #4, n=1) and non-responders before (Blood draw #1, n=11; Blood draw #2, n=8; Blood draw #3, n=10, Blood draw #4, n=4); one-way ANOVA using Dunnett's multiple comparison test. (**B**) The frequency of influenza Th1-like(EM CXCR3+) cells in Responders before (Blood draw #1, n=9) and after therapy (Blood draw #2, n=5; Blood draw #3, n=5; Blood draw #4, n=1) and non-responders before (Blood draw #1, n=11; Blood draw #2, n=8; Blood draw #3, n=10, Blood draw #4, n=4); one-way ANOVA using Dunnett's multiple comparison test. For A and B datapoints are those samples where parent event count is at least 8.

Supplemental Table 1. Tetramer panel used for *ex-vivo* analysis of cross-sectional cohort

Epitope pool	Epitopes	Tetramer Color	Sequences
Enolase	Eno 11-25	PE	IFDS (CIT) GNPTVEVDLF
	Eno 26-40		TSKGLF (CIT) AAVPSGAS
	Eno 326-340		K (CIT) IAKAVNEKSCNCL
VimFib	Vim 59-78	PE-Cy5	GVYAT (CIT) SSAV (CIT) L (CIT) SSVPGVR
	Vim 418-431		SSLNL (CIT) ETNLDSL
	Fib A 24-38		EGDFLAEGGGV (CIT) GPR
	Fib B 69-81		GY (CIT) A (CIT) PAKAAAT
CILP	CILP 297-311	PE-CF594	ATIKAEFV (CIT) AETPYM
	CILP 982-986		GKLYGI (CIT) DV (CIT) STRDR
Aggrecan	Agg 225-244	APC	DEFPGV (CIT) TYGI (CIT) DTNETYDV
	Agg 553-570		PGV (CIT) TYGV (CIT) PSTETYDVY
Influenza	CaHa 265-284	BV421	LVVPRYAFAMERNAGSGIII
	TxHa 321-340		CPRYVKQSTLKLATGMRNVP

Supplemental Table 2. Antibodies used for *ex-vivo* tetramer analysis of cross-sectional cohort

Marker	Format	Clone	Manufacturer
CD14	FITC	HCD14	Biolegend
CD19	FITC	HIB19	Biolegend
Dead	FITC	n/a	Thermo Fisher
CD4	V500	RPA-T4	BD Biosciences
CD45RA	AF700	HI100	BD Biosciences
CCR7	PerCP/Cy5.5	G043H7	Biolegend
CXCR3	PE/Cy7	G025H7	Biolegend
CCR4	BV605	L291H4	Biolegend
CCR6	BV786	G034E3	Biolegend
CD38	BUV395	HB7	BD Biosciences

Supplemental Table 3. Antibodies used for metacluster confirmation

Marker	Format	Clone	Manufacturer
CD14	FITC	HCD14	Biolegend
CD19	FITC	HIB19	Biolegend
Dead	FITC	n/a	Thermo Fisher
CD4	V500	RPA-T4	BD Biosciences
CD45RA	AF700	HI100	BD Biosciences
CCR7	PerCP/Cy5.5	G043H7	Biolegend
CXCR3	PE/Cy7	G025H7	Biolegend
CCR4	BV605	L291H4	Biolegend
CCR6	BV786	G034E3	Biolegend
CD38	BUV395	HB7	BD Biosciences
IFNγ	APC/Cy7	4S.B3	Biolegend
IL-4	PE	8D4-8	Biolegend
IL-17	A647	BL168	Biolegend

Supplemental Table 4. Samples eligible for DISCOVR analysis

Number of samples with at least 8 events for DISCOVR analysis								
	Total	CD4T	Influenza	Cit-RA	Aggrecan	CILP	Enolase	Vim&Fib
Healthy	27	27	27	27	17	21	21	13
RA	64	64	61	63	42	35	36	36

Supplemental Table 5. Longitudinal cohort demographics and clinical characteristics[&]

Demographics and clinical characteristics	Active RA (n=25)	Quiescent RA (n=12)
**Median age in years at blood draw (Range: min-max)**	62 (29-89)	63 (28-77)
**Male/Female (number of subjects)**	8/17	6/6
**Disease duration in years (mean±sd)**	11.3 ± 10.2	14.5 ± 13.1
**Disease activity based on weighted RAPID3 score* (number of subjects)**	**wRAPID3 >2.3 (n=25)**	**wRAPID3 <2.0 (n=12)**
Near remission (0 – 1.0)	0	8
Low activity (1.3 – 2.0)	0	4
Moderate activity (2.3 – 4.0)	10	0
High activity (4.3 – 10.0)	15	0
**Therapy at baseline blood draw** (number of subjects (% of cohort))**		
Steroids	11 (41%)	4 (33%)
DMARDS	13 (52%)	8 (67%)
Biologics	3 (12%)	2 (17%)
**Type of biologic at baseline blood draw (number of subjects)**		
TNF inhibitor***	2	2
JAK inhibitor (tofacitinib)	1	0
**New therapy at second blood draw** (number of subjects (% of cohort))**		
DMARDs	6 (24%)	3 (25%)
Biologics	17 (68%)	7 (58%)
Biologic label change (TNF inhibitor to TNF inhibitor)	2 (8%)	2 (17%)
**Met criteria for tmr analysis[&] (number of subjects)**	20	0
**Number of samples**	74	34

[&] Inclusion criteria for tetramer analysis: active RA at initial blood draw with weighted RAPID3 greater than 2.3; new therapy at second blood draw, both initial and second blood draw pass tetramer influenza quality control

* RAPID3, routine assessment of patint index data with 3 measures (0-10)

**Therapy at baseline blood draw: Steroids include prednisone not more than 10mg/day within 1 week of blood draw; DMARDs include azathioprine, hydroxychloroquine, leflunomide, methotrexate and sulfasalazine

***TNF inhibitors include adalimumab, etanercept, golimumab and infliximab

Supplemental Table 6. Tetramer panel used for *ex-vivo* analysis of longitudinal cohort

DISCOVR	Antigen	Position (aa #)	Tetramer Color	Sequence
	alpha-Enolase	11-25	PE	IFDS (CIT) GNPTVEVDLF
	alpha-Enolase	26-40	PE	TSKGLF (CIT) AAVPSGAS
	alpha-Enolase	326-340	PE	K (CIT) IAKAVNEKSCNCL
X	Vimentin	59-78	PE-Cy5	GVYAT (CIT) SSAV (CIT) L (CIT) SSVPGVR
X	Vimentin	418-431	PE-Cy5	SSLNL (CIT) ETNLDSL
X	alpha-Fibrinogen	24-38	PE-Cy5	EGDFLAEGGGV (CIT) GPR
X	beta-Fibrinogen	69-81	PE-Cy5	GY (CIT) A (CIT) PAKAAAT
	Tenascin-C	871-885	PE-CF594	VSLILS (CIT) (CIT) GDMSSNPA
	Tenascin-C	1816-1830	PE-CF594	PDGF (CIT) LSWTADEGVF
	Tenascin-C	2250-2264	PE-CF594	QGQYEL (CIT) VDL (CIT) DHGE
X	Aggrecan	225-244	APC	DEFPGV (CIT) TYGI (CIT) DTNETYDV
X	Aggrecan	553-570	APC	PGV (CIT) TYGV (CIT) PSTETYDVY
X	Influenza	97-116	BV421	VKLYRKLKREITFHGAKEIS

Supplemental Table 7. Antibodies used for *ex-vivo* analysis of longitudinal cohort

DISCOVR	Marker	Format	Clone	Manufacturer
	CD14	FITC	HCD14	Biolegend
	CD19	FITC	HIB19	Biolegend
	Dead	FITC	n/a	Thermo Fisher
	CD4	V500	RPA-T4	BD Biosciences
X	CD45RA	AF700	HI100	BD Biosciences
X	CCR7	APC-Cy7	G043H7	Biolegend
X	CXCR3	PE/Cy7	G025H7	Biolegend
X	CCR4	BV605	L291H4	Biolegend
X	CCR6	BV786	G034E3	Biolegend
	CD25	BV711	2A3	BD Biosciences
X	CD38	BUV395	HB7	BD Biosciences
	LAG3	BV650	11C3C65	Biolegend
	PD1	BUV737	EH12.1	BD Biosciences
	CXCR5	BB700	RF8B2	BD Biosciences
